# A Many-Faced Alkaloid: Polymorphism of (–)-Monophyllidin

**DOI:** 10.3390/molecules25030449

**Published:** 2020-01-21

**Authors:** Christian Dank, Richard Wurzer, Susanne Felsinger, Ricarda Bugl, Hanspeter Kählig, Wolfgang Hela, Alexander Roller, Hubert Gstach

**Affiliations:** 1 Institute of Organic Chemistry, Faculty of Chemistry, University of Vienna, Waehringer Strasse 38, 1090 Vienna, Austria; a0307871@univie.ac.at (R.W.); hanspeter.kaehlig@univie.ac.at (H.K.); 2 NMR Centre, Faculty of Chemistry, University of Vienna, Waehringer Strasse 38, 1090 Vienna, Austria; susanne.felsinger@univie.ac.at (S.F.); ricarda.bugl@univie.ac.at (R.B.); 3 Medicinal Chemistry, Boehringer Ingelheim RCV GmbH & Co KG, Dr.-Boehringer-Gasse 5-11, 1121 Vienna, Austria; wolfgang.hela@boehringer-ingelheim.com; 4 Institute of Inorganic Chemistry, Faculty of Chemistry, University of Vienna, Waehringer Strasse 42, 1090 Vienna, Austria; 5 Department of Pharmaceutical Chemistry, Faculty of Life Sciences, University of Vienna, UZ2 E349, Althanstrasse 14, 1090 Vienna, Austria

**Keywords:** natural product, monophyllidin, zanthoxylum, alkaloid, polymorphism, hydrogen bonding, chelate.

## Abstract

The synthesis of the alkaloid (–)-monophyllidin is described. The molecule is a hybrid of xanthoxyline and (*S*)-proline, accessible in one-step through a Mannich reaction. In the solid-state, defined structural arrangements with different physical properties are formed. Single crystal X-ray diffraction revealed structures of six distinct polymorphs. In the crystalline state, the alkaloid can host small polar molecules (preferably water), while the (*S*)-proline moiety is present in the zwitterionic state. Combined with the chelate, which is already present in the xanthoxyline substructure, an ideal disposition for multiple hydrogen bond networks evolve. Therefore, highly water-soluble polymorphs of monophyllidin can form. This structural flexibility explains the many faces of the molecule in terms of structure as well as analytical data. Furthermore, speculations about the biological role of the molecule, with regard to the manifold interactions with water, are presented.

## 1. Introduction

**Scheme 1:**
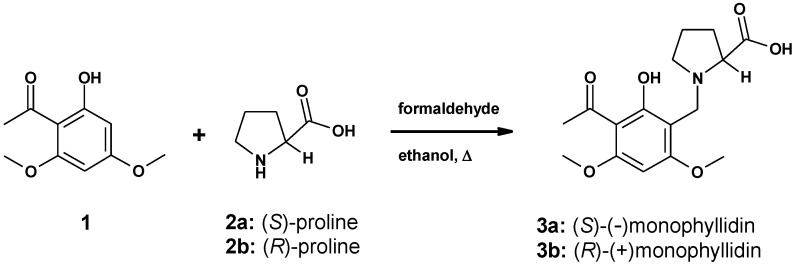
Synthesis of (–)-monophyllidin (**3a**) and (+)-monophyllidin (**3b**).

(–)-Monophyllidin (**3a**) is a natural alkaloid isolated from the bark of *Zanthoxylum* *monophyllum* [1]. The species of *Z. monophyllum* is endemic in Middle and South America as well as the Caribbean [2]. Species of the genus *Zanthoxylum* have been used in traditional medicine for the treatment of disorders and also play a role in alimentary and industrial commodities [3,4]. Therefore, *Z. monophyllum* is considered as a promising source for new biologically active alkaloids.

(–)-Monophyllidin (**3a**) is a hybrid of two natural products, xanthoxyline (**1**) and (S)-proline (**2a**), respectively (Scheme 1). Xanthoxyline (**1**) is also a widespread natural compound isolated from numerous diverse sources of plants. The structure of (–)-monophyllidin (**3a)** was elucidated through spectroscopic data analysis. The absolute configuration was derived from the negative rotation and attributed to an (*S*)-proline moiety by comparison with lit. values [1,5].

We describe an efficient one -step synthesis of (–)-monophyllidin (**3a**) and (+)-monophyllidin (**3b**) (Scheme 1). The analytical characterization of **3a** reveals striking differences in physical parameters like melting point, optical rotation, as well as IR-spectrum [1]. Only the NMR-spectra were in good agreement. The melting point was found to be 10 °C higher; the IR-spectrum did not reveal any absorption in the range of 1700–1800 cm^−1^ and the optical rotation was found to be much lower than reported. We can confirm the molecular structure reported by Patino and Cuca [1]. However, the obvious differences found in the physical properties demand an explanation and point towards an overseen structural characteristic of (–)-monophyllidin (**3a)**. In the following sections, we describe the reasons for the found discrepancies and show that this small natural alkaloid has many faces indeed. The structural flexibility of **3a** may be related to a biological function of this intriguing compound.

## 2. Results and Discussion

### 2.1. Synthesis of (–)- and (+)-Monophyllidin 

(–)-Monophyllidin (**3a**) was accessible by linking xanthoxyline (**1**) to (S)-proline (**2a**) in a one-step Mannich reaction (Scheme 1). The reaction is regiospecific for the sterically less hindered 3-position in xanthoxyline (**1**). (+)-Monophyllidin (**3b**) was synthesized analogously starting from (*R*)-proline (**2b**).

The pure material of **3a** obtained after chromatographic separation of the reaction mixture revealed a melting point of 157–158 °C (under decomposition), which was significantly higher than the reported value of 150–151 °C. An even more significant deviation was found for the specific optical rotation: ${\left[\alpha \right]}_{20}^{D}$ = −12.5° [c = 1.00, CHCl_3_)] and ${\left[\alpha \right]}_{20}^{D}$ = −8° [c = 0.28, CHCl_3_] for synthesized **3a** compared to ${\left[\alpha \right]}_{20}^{D}$ = −40.3° (c = 0.28, CHCl_3_) from literature. Also, the IR-spectrum of **3a** (KBr) did not reveal any absorption in the range of 1700–1800 cm^−1^.

### 2.2. Synthesis of the Ester of (–)-Monophyllidin and (+)-Menthol

To address first the deviation in the optical rotation, we checked the stability of the (*S*)–proline (**2a**) stereocenter in the Mannich reaction. For this purpose, we synthesized a (1*S*, 2*R*, 5*S*)-menthyl ester of (*S*)-proline (**4**) [6], which could be subjected to single-crystal X-ray diffraction analysis as hydrochloride (**4xHCl**).

**Scheme 2:**
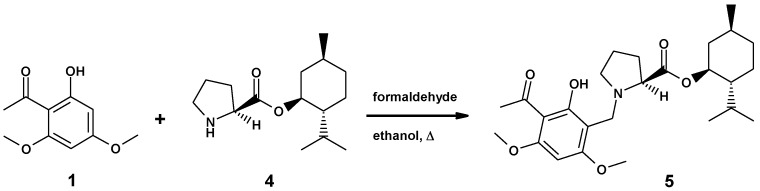
Synthesis of (–)-monophyllidin-(+)-menthyl ester **5**.

The enantiomerically pure **4xHCl** was reacted with xanthoxyline (**1**) to afford menthyl ester **5** (Scheme 2). A single stereoisomer of monophyllidin menthyl ester **5** was obtained as indicated by NMR. Only one set of ^13^C-resonances was recorded. This result suggested already that the configuration of (S)–proline remained stable under the Mannich reaction conditions. Compound **5** was transformed to crystalline hydrochloride **5xHCl**. The single-crystal X-ray structure of **5xHCl** confirmed the absolute configuration of the stereocenters in **5**, including the questioned stereocenter of the unaltered (*S*)-proline residue (for X-ray, see chapter 3.6).

Additionally, the enantiomeric purities of (–)-monophyllidin (**3a**) and (+)-monophyllidin (**3b**) obtained from the Mannich reactions were determined by chiral supercritical fluid chromatography (SFC). **3a** revealed an enantiomeric excess (ee) > 98%, with a t_R_ = 2.4 min whereas **3b** showed a lower ee of 93% with t_R_ = 3.3 min (for chromatograms see Supplementary Materials). The difference in the enantiomeric access between the two synthesized enantiomers **3a** and **3b** can be attributed to the differences in optical purity of the commercial (*R*)- and (*S*)-prolines used as starting materials. The stability of the stereocenter during synthesis is proven, as described above. The optical rotation was found in good agreement between the two enantiomers (−12.5° for **3a** vs. +11.2° for **3b**), suggesting a correction of the reported literature value for (–)- monophyllidin **3a**.

To find an answer for the other observed deviations in physical parameters, we proceeded with crystallographic investigations. Inspection of the crystal charge of **3a** obtained from the chromatographic separation (primary crystals; mp 157–158 °C) revealed already a mixture of crystal forms. We could identify block-like crystals and tiny needles as well as some plates, indicating that (–)-monophyllidin may be prone to polymorphism. Therefore, we gave focus to the crystallization of **3a** and found a couple of solvents supporting crystal growth of **3a.**

Recrystallization of **3a** (primary crystals) from acetonitrile afforded three different crystal forms: mainly block-like crystals hosting only acetonitrile (polymorph **P-I**), tiny needles containing acetonitrile and water (polymorph **P-II**), and plates hosting only water (polymorph **P-VI**). The latter crystal appeared as a singleton, but nevertheless, X-ray data could be collected. Crystals grown from chloroform revealed needles were hosting chloroform and water (polymorph **P-III**). Crystallization of **3a** from ethanol yielded needle-shaped crystals not suitable for analysis. However, subsequent recrystallization from acetonitrile produced solely big needle-shaped crystals containing only water (polymorph **P-IV**). Finally, block-like crystals of **3a** formed in water upon cooling (polymorph **P-V**). Crystals of **P-V** host water. Melting points and space groups of identified polymorphs **P-I** to **P-VI** are summarized in Table 1.

The results of the single-crystal X-ray structure analysis of the distinct (–)-monophyllidin polymorphs (**P-I** to **P-VI**) are presented in the following Section 3.1, Section 3.2, Section 3.3, Section 3.4, Section 3.5 and Section 3.6 (experimental parameters, CCDC-codes, sample and crystal data, data collection, structure refinement and hydrogen bond geometries of the polymorphs are provided in the Supplementary Materials). The X-ray analysis confirmed the molecular structure of (–)-monophyllidin **3a** as well as the *S*-configuration of the proline stereocenter.

## 3. Crystallographic Characterization of (–)-Monophyllidin Polymorphs (P-I to P-VI)

### 3.1. (–)-Monophyllidin Polymorph-I (P-I) (Crystallized from CH_3_CN; Space Group P2_1_2_1_2_1_; Crystal System Orthorhombic)

**Figure 1:**
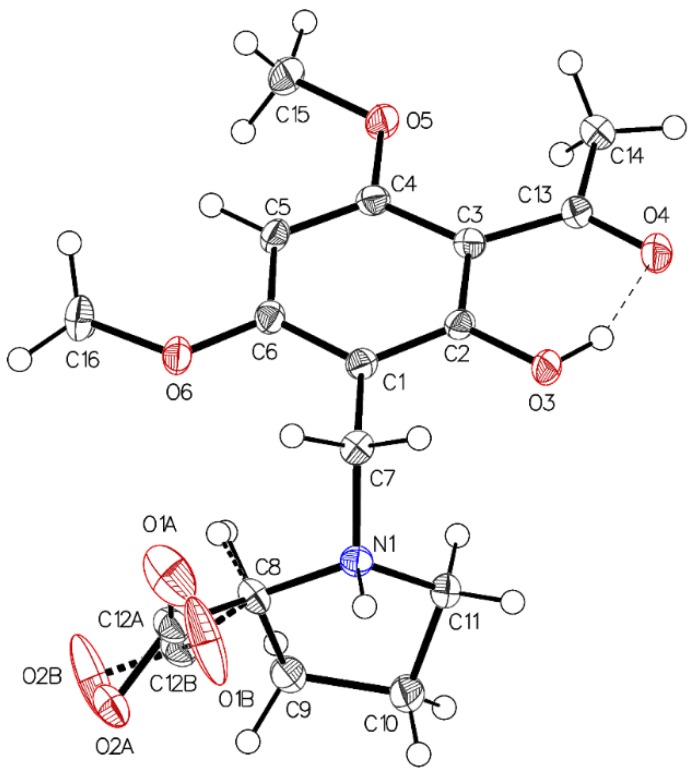
The asymmetric unit of **P-I**, drawn with 50% displacement ellipsoids. The main residue disorder is 13%. It is interpreted as two different possible conformations caused by short contacts between the carboxylate oxygens (acetonitrile not shown for clarity reason).

**Figure 2:**
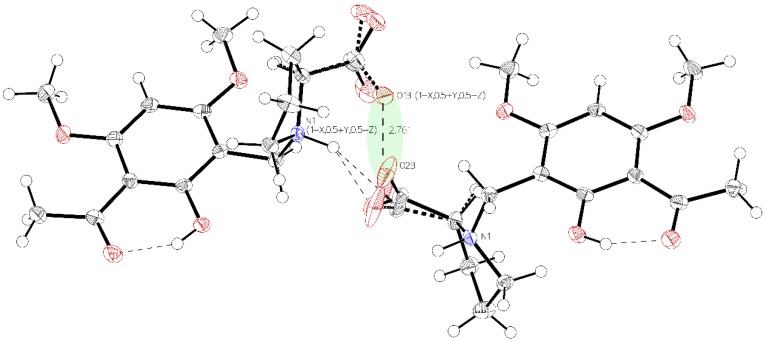
Visualization of the very close oxygen interaction O1B to O2B (light green). Due to charge repulsion, Part A and Part B must alternate their positions.

Single crystal X-ray diffraction analysis of the higher melting (161–162 °C) block-like crystals obtained from the recrystallization of **3a** in acetonitrile (polymorph **P-I**) revealed one molecule in the asymmetric unit with proline in a zwitterionic state. The carboxylate residue shows a disorder (Figure 1). The phenolic group is involved in intramolecular hydrogen bonding to the acetyl carbonyl (aryl-OH•••O=C(CH_3_). The crystals accommodate acetonitrile but no water molecules. The disorder of the carboxylate is most probably caused by close contact (2.7 Å) of the negatively charged carboxylates in the crystal. The interaction is depicted in Figure 2.

### 3.2. (–)-Monophyllidin Polymorph-II (P-II) (Crystallized from CH_3_CN; Space Group C2; Crystal System Monoclinic)

**Figure 3:**
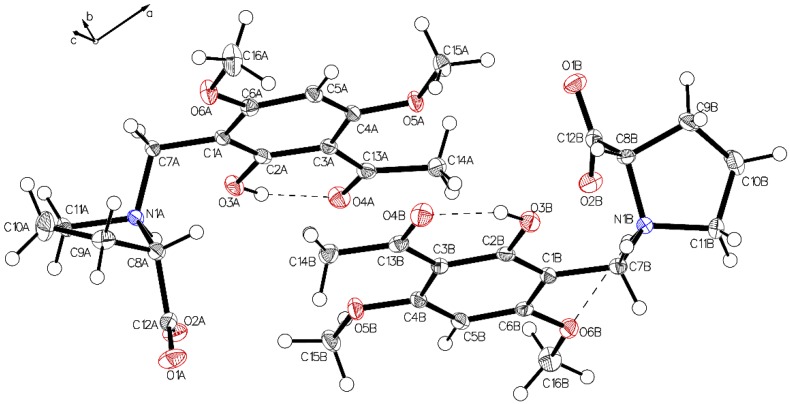
The asymmetric unit of **P-II** drawn with 50% displacement ellipsoids, containing two independent molecules (molecule **A**, left; molecule **B**, right) in different hydrogen bonding modes.

**Figure 4:**
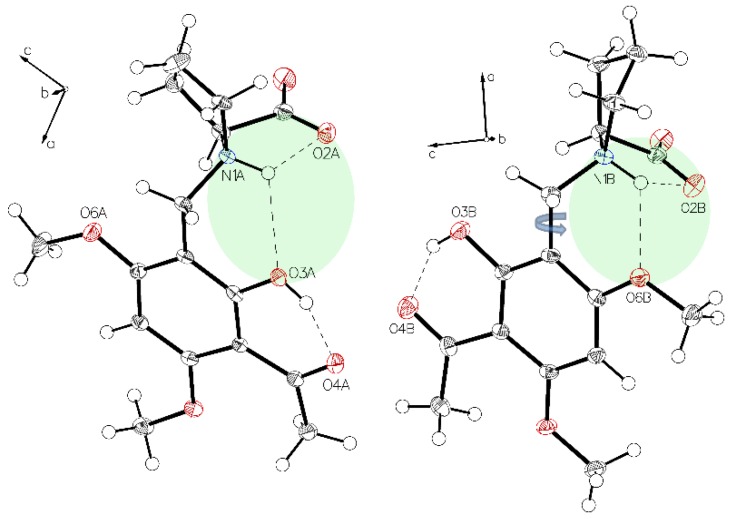
The two hydrogen bonding modes in (–)-monophyllidin polymorph **P-II**. Bifurcated N^+^H interaction to carboxylate oxygen O2A and phenolic oxygen O3A in molecule **A** (left) is disrupted upon a rotation along the C1–C7 bond enabling the formation of an alternative hydrogen bond between N^+^H and oxygen O6 of the methoxy group on the xanthoxyline residue (molecule **B**, right).

The asymmetric unit of **P-II** contains two independent molecules. The proline part is uniformly in a zwitterionic state in both molecules. In molecule **A,** the protonated proline nitrogen forms bifurcated hydrogen bonding to the carboxylate function and to the phenolic oxygen of the intramolecular chelate present in the acetophenone moiety (Figure 3, left). Whereas in molecule **B,** the corresponding N^+^H bifurcates the hydrogen bonding to the oxygen of a methoxy group instead of the phenolic oxygen in molecule **A** (Figure 3, right). This interaction is achieved by a rotation close to 180° along the C1–C7 bond (Figure 4). The crystals accommodate acetonitrile and water.

**Figure 5:**
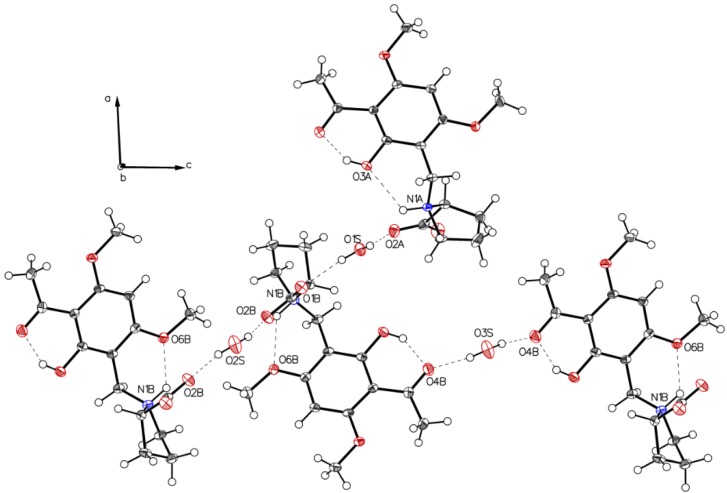
Crystal packing of **P-II**. Water molecules (depicted O2S and O1S) connect molecules through interaction with the carboxylate groups. Further stabilization of the crystal is mediated by water molecules (depicted O3S), which serve as double hydrogen-donors such connecting two neighboring molecules through the acetyl oxygens (O4B-HOH-O4B).

The crystal packing of **P-II** is depicted in Figure 5.

### 3.3. (–)-Monophyllidin Polymorph-III (P-III) (Crystallized from CHCl_3_; Space Group P1; Crystal System Triclinic)

**Figure 6:**
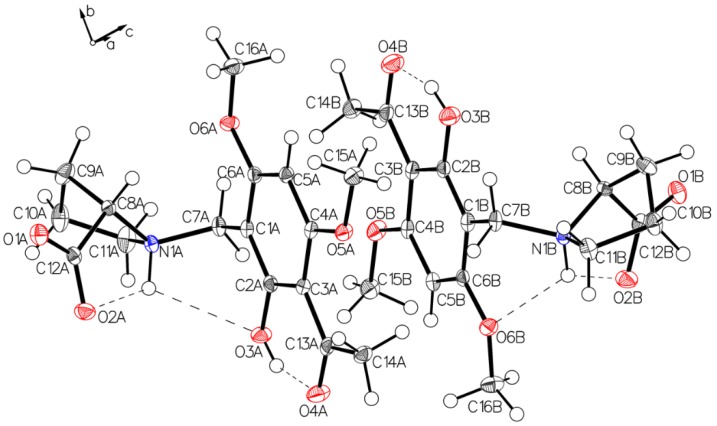
The asymmetric unit of **P-III**, drawn with 50% displacement ellipsoids. The solvents chloroform and water are hidden for clarity—Molecule **A** on the left, molecule **B** on the right.

**Figure 7:**
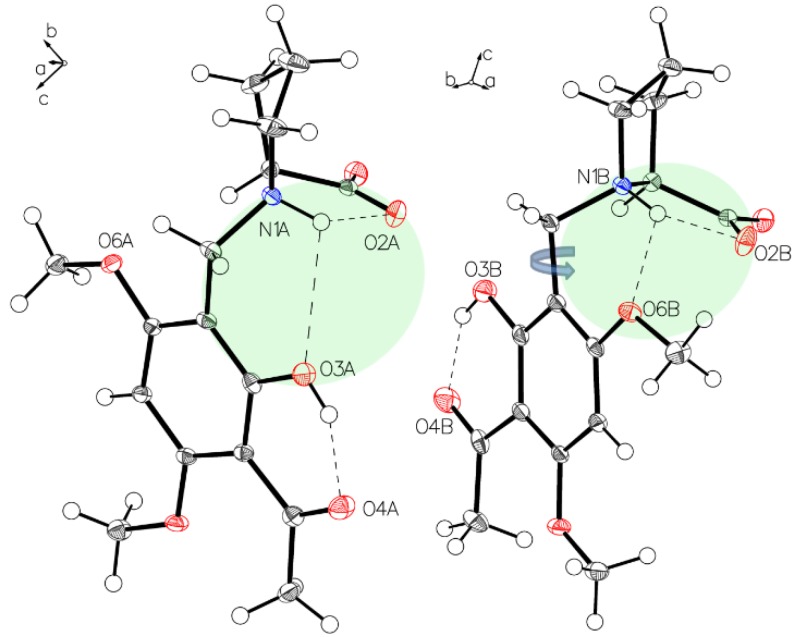
The main difference between the two independent molecules of **P-III** in the asymmetric unit is the rotation close to 180° along the C1–C7 bond (blue arrow). Short interaction N1A-H O2A, N1A-H O3A for molecule **A** (left) and N1B-H O2B, N1B-H O6B for molecule **B** (right) affect the structures of the two molecules.

The asymmetric unit of **P-III** contains two independent molecules which form different intramolecular hydrogen bonds regarding to the zwitterionic proline residue. In molecule **A,** the protonated proline nitrogen forms bifurcated hydrogen bonding to the carboxylate function and to the phenolic oxygen of the intramolecular chelate (Figure 6, left). Whereas in molecule **B,** the corresponding N^+^H bifurcates the hydrogen bonding to the oxygen of a methoxy group instead of the phenolic oxygen in molecule **A** (Figure 6, right). This interaction is achieved by a rotation along the C1–C7 bond (Figure 7). The crystals accommodate chloroform and water molecules.

**Figure 8:**
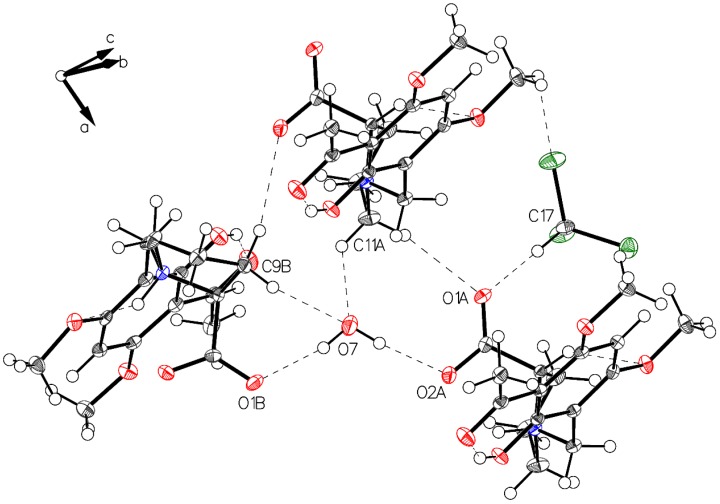
Visualization of water connected to carboxylate oxygens (O1B–O7–O2A). Also, visualized weak interactions form water to hydrogens located on C9B and C11A and the interaction O1A to the acidic hydrogen on C17 of the solvent CHCl_3_.

Crystal packing of **P-III** is depicted in Figure 8. A water molecule bridges the two carboxylates of molecule **A** and molecule **B**.

### 3.4. (–)-Monophyllidin Polymorph-IV (P-IV) (Obtained from Recrystallization in Ethanol Followed by Acetonitrile; Space Group P1; Crystal System Triclinic)

**Figure 9:**
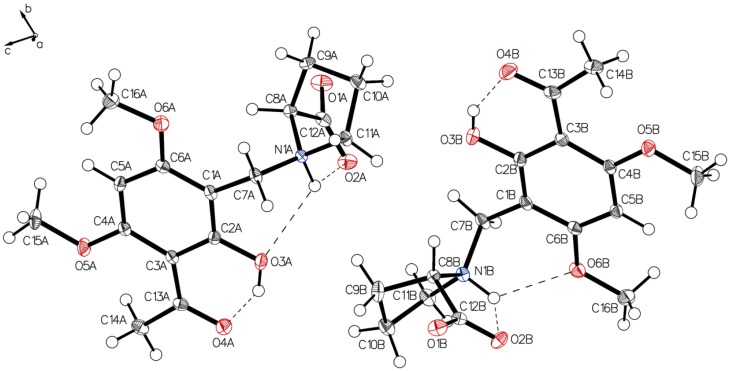
Two independent molecules form the asymmetric unit of **P-IV**, drawn with 50% displacement ellipsoids (water molecules hidden for clarity).

**Figure 10:**
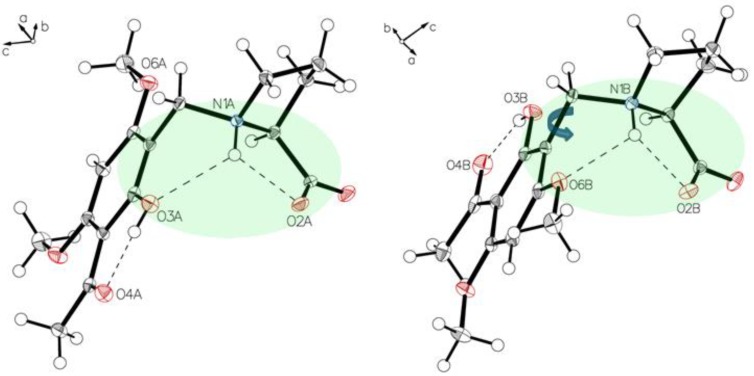
The main difference in two independent molecules, **A** (left panel) and **B** (right panel) of **P-IV** in the asymmetric unit is the rotation along C1–C7. Short interactions N1A-H O2A, N1A-H O3A for molecule **A** (left) and N1B-H O2B, N1B-H O6B for molecule **B** (right) affect the structures of the two molecules.

Analysis of the needles of **3a** (**P-IV)** revealed the proline part of (–)-monophyllidin (**3a**) also in a uniform zwitterionic protonation state. The crystals of **P-IV** contain a lot of water (in contrast to **P-III**). The carboxylate residues of the proline interact mediated by water molecules. The protonated proline-nitrogen was found in two different interactions, as already observed in crystals of **P-II** and **P-III**. These interactions create two independent molecules, **A** and **B,** in the asymmetric unit. In molecule **A** (Figure 9 and Figure 10; left), the N^+^H forms bifurcated hydrogen bonding between carboxylate oxygen and a phenolic group of the chelate. Molecule **B** (Figure 9 and Figure 10; right) also reveals a bifurcated hydrogen bonding of the N^+^H, but also shows a rotation around the C1–C7 bond compared to molecule **A** resulting in a hydrogen bond to the O6B of a methoxy group on the acetophenone moiety. The hydrogen bond between phenolic hydrogen and the carbonyl oxygen of the acetyl group forms the intramolecular chelate in both molecules **A** and **B**.

**Figure 11:**
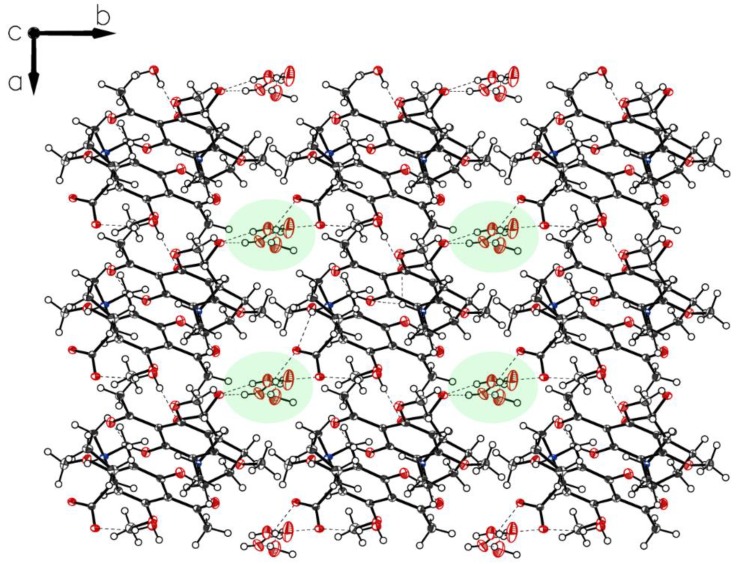
Packing of **P-IV**. Along c, a cylindric void packed with ordered water molecules appears.

**Figure 12:**
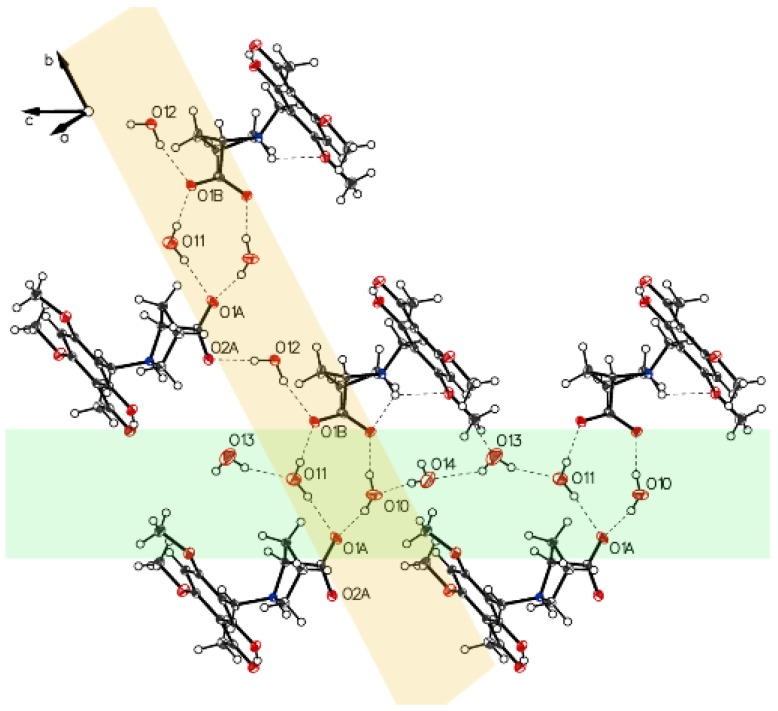
Molecules bridged by hydrogen bonds (water and carboxylate of P**-IV** build up chains, along axes b and c).

More interactions governing the crystal of **3a**-polymorph **P-IV** can be gained by an inspection of the crystal packing. **P-IV** crystals contain water molecules in a highly ordered state resulting in water channels through the c-axis of the crystal stabilized by surrounding carboxylates and separated by hydrophobic borders (Figure 11). Carboxylates and water form chains along with axes b and c (Figure 12).

### 3.5. (–)-Monophyllidin Polymorph-V (P-V) (Obtained from Water; Space Group P2_1_; Crystal System Monoclinic)

**Figure 13:**
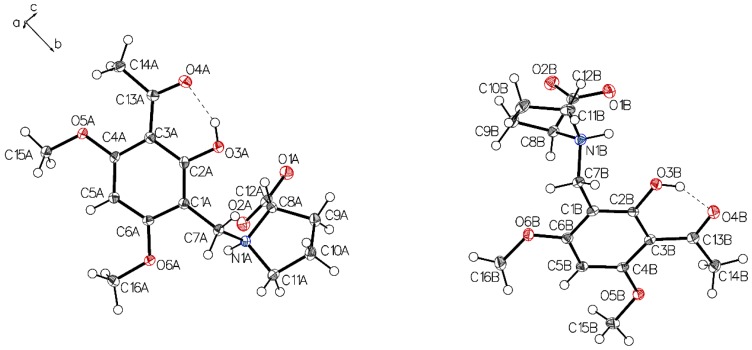
An asymmetric unit of **P-V**, drawn with 50% displacement ellipsoids. Solvent omitted for clarity (molecule **A**, left; molecule **B**, right).

**Figure 14:**
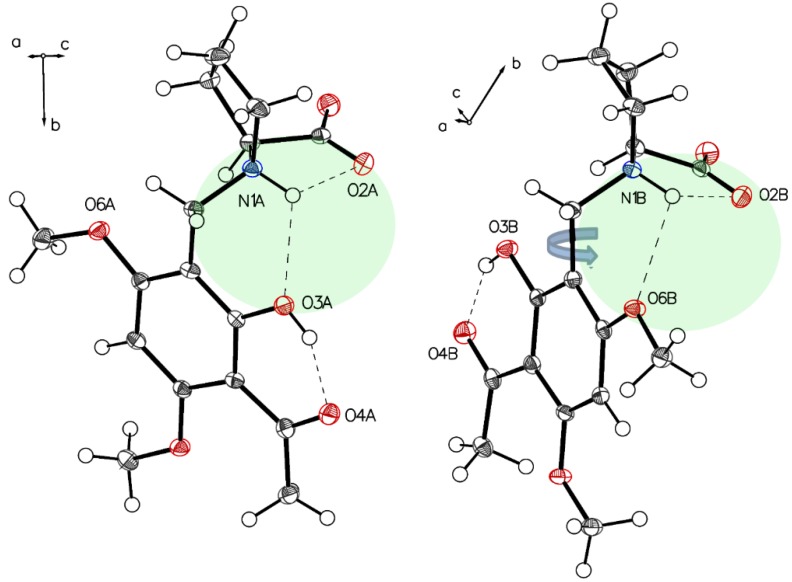
The main difference in two independent molecules of **P-V** in the asymmetric unit is the rotation close to 180° along C1–C7. Short interaction N1A-H O2A, N1A-H O3A for molecule **A** (left) and N1B-H O2B, N1B-H O6B for molecule **B** (right) affect structures of the two molecules.

**Figure 15:**
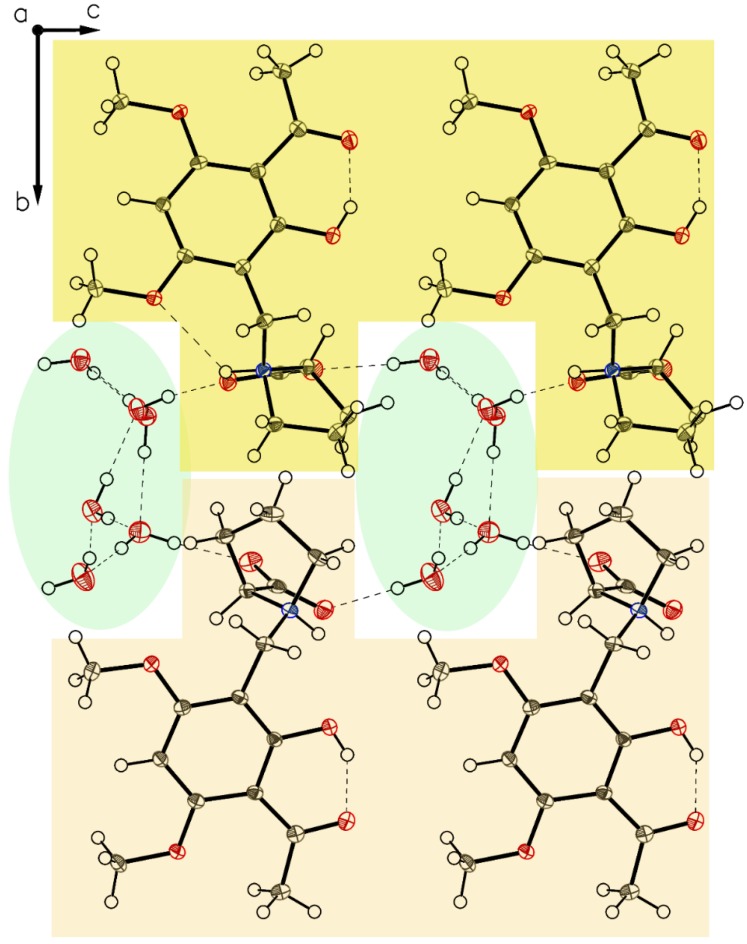
Packing of **P-V**. The two different layers of molecules **A** and **B** characterized by light yellow on top, and light orange on the bottom along axis b enclose the three molecules of co-crystallized water each. Hydrogen bonded molecular aggregates connect all molecules through the water.

Crystals of **P-V** grown from water show the proline part also in a zwitterionic state. Two independent molecules **A** and **B** are present, which are in different hydrogen-bonding modes as also found for **P-II** to **P-IV** (Figure 13 and Figure 14). The crystal packing of **P-V** reveals a design formed by alternating layers of molecules **A** and **B** (Figure 15). Highly ordered aggregates connect all the molecules through the water.

### 3.6. (–)-Monophyllidin Polymorph-VI (P-VI) (Plate Isolated from the Primary Crystal Mixture; Space Group P2_1_2_1_2_1_; Crystal System Orthorhombic)

**Figure 16:**
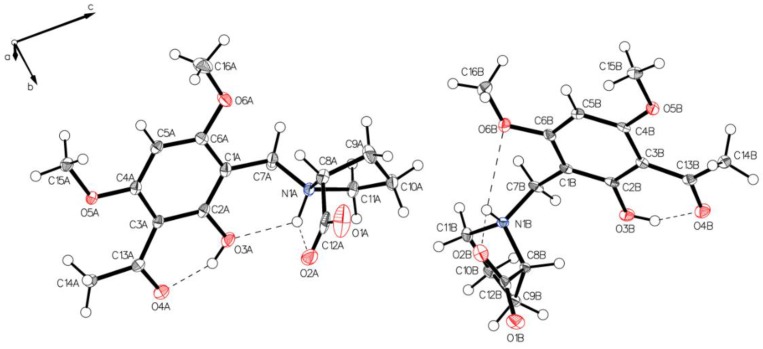
The asymmetric unit of **P-VI**, drawn with 50% displacement ellipsoids. Solvent is hidden for clarity. A rotation close to 180° along C1–C7 and its consequences for hydrogen bonds is the main difference for the two independent molecules (molecule **A**, left; molecule **B**, right).

**Figure 17:**
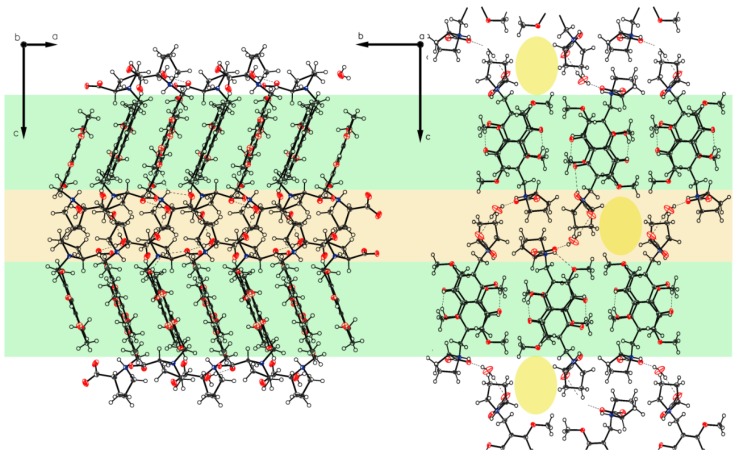
Packing of **P-VI**. The two-layered design distinguishes between hygroscopic (light orange) and non-hygroscopic (light green) areas in planes ac and bc. Also visualized by yellow elliptic areas not exact identified solvent void characterized by the Olex2 mask tool (V = 105.6 Å^3^, e^−^ = 29.2). Again, as for **P-IV**, the main direction for hydrogen bond bridges can be identified (along axis c).

The structure of polymorph **P-VI** reveals the proline moiety also in a defined zwitterionic state with the proline nitrogen atom protonated and the carboxyl residue deprotonated to a carboxylate anion. The unit cell contains two independent molecules **A** and **B** in different H-bonding modes (Figure 16), as found for **P-II** to **P-V**. In both molecules, the intramolecular chelate between phenolic OH as a donor and the carbonyl oxygen of the acetyl group as an acceptor is established. The crystal contains one ordered water molecule which connects two carboxylates of molecule **A** and molecule **B**. Crystal packing leads to a layered crystal architecture with significant voids in the lattice. These voids can serve as hosts for additional small guest molecules, e.g. for further water molecules (Figure 17).

### 3.7. (S)-(1S,2R,5S)-2-Isopropyl-5-methylcyclohexyl1-(3-acetyl-2-hydroxy-4,6-dimethoxy-benzyl) pyrrolidine-2-carboxylate Hydrochloride (5xHCl).

**Figure 18:**
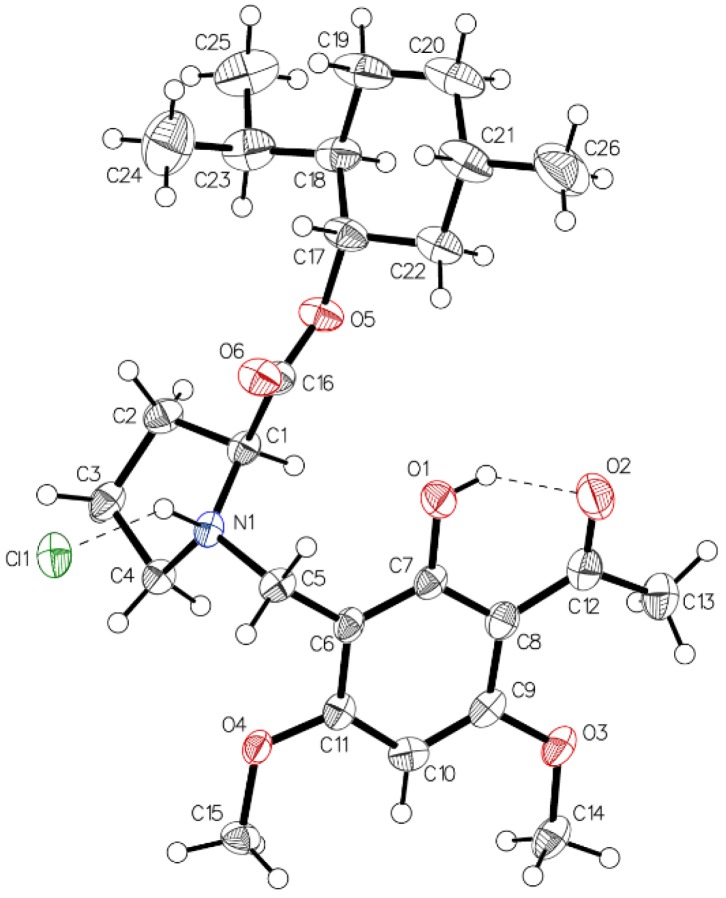
The asymmetric unit of **5xHCl**, drawn with 50% displacement ellipsoids. Two main interactions influence the system. One intramolecular (O1–O2) hydrogen bond of moderate character and one intermolecular (N1^+^−Cl1−) which expresses the ionic character of the molecule. Chirality determination proved by the Hooft (0.02(4)) and the Flack (0.10(4)) parameter only off grade.

Finally, crystals of the hydrochloride of (–)-monophyllidin (1*S*,2*R*,5*S*)-menthyl ester (**4**) suitable for X-ray diffraction analysis could be grown. The structure proves the unaltered configuration of the stereogenic center in the proline moiety. Figure 18 shows the two main interactions predominant in **5xHCl**. An intramolecular (O1–H•••O2) hydrogen bond of strong character and an intermolecular (N1–H•••Cl1) interaction, which expresses the ionic character of the molecule. Chirality determination is proofed by Hooft (0.02(4)) and Flack (0.10(4)) parameter in acceptable quality. No crystal could be obtained from the corresponding free base **5**. 

**Figure 19:**
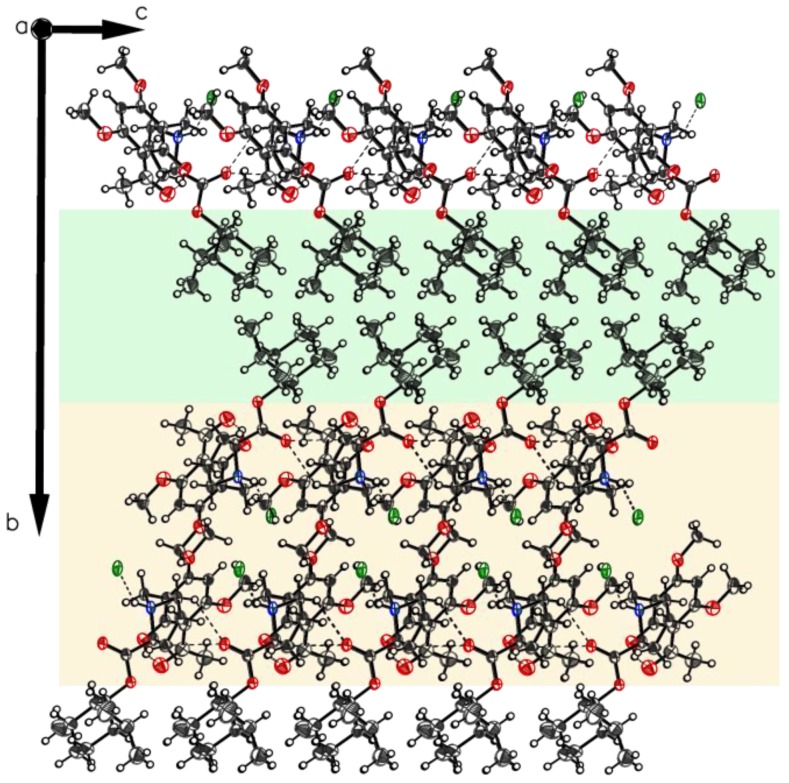
Packing of **5xHCl** in the plane bc. The two-layered design which differentiates between ionic (orange shaded) and non-ionic (green shaded) interactions.

Crystal packing of **5xHCl** (Figure 19) reveals a two-layered design that differentiates between parts governed by ionic (orange shaded) and non-ionic (green shaded interactions). The plate morphology of the crystal and the long b axis in comparison to the axes a and c are in good accordance with common crystal growth. Only one hydrogen bonding modus is visible. The chelate is not assisted by the N^+^H-donor, which is solely involved in *Coulombic* interaction to the chloride ion.

### 3.8. Summary and Conclusions

The results demonstrate the difficulty of obtaining a classical characterization of (–)-monophyllidin through physical methods, such as melting point determination. The latter turned out to be a function of chemical parameters applied in the isolation and purification procedures of the molecule.

The crystallographic characterization of (–)-monophyllidin (**3a**) disclosed a polymorphism of the molecule. Six polymorphs could be characterized by single-crystal X-ray crystallography in our examinations, and even more, may exist. The polymorphs cover four different space groups. None of the crystals investigated contained only the target molecule **3a**. All crystals hosted guest molecules in different compositions. In five examples, water was present, either together with an organic solvent or as the sole guest molecule (three examples). Recrystallization of **3a** from acetonitrile produced three polymorphs (**P-I**, **P-II,** and **P-VI**) covering two space groups. Crystals of **P-I** (space group *P2_1_2_1_2_1_*) were the only example where no water co-crystallized. **P-II** (space group *C2*) contained acetonitrile and water, and **P-IV** (space group *P2_1_2_1_2_1_*) solely water, respectively. Crystals grown from chloroform revealed an additional polymorph **P-III** (space group *P1*), hosting organic solvent and water. The remaining polymorphs **P-IV** (space group *P1*) and **P-V** (space group *P2_1_*) accommodate only water molecules in a highly ordered state. The results from the crystallographic investigations are summarized in Table 2.

In all the solved structures, the proline residue was found in a zwitterionic state. Also common to all the structures was the presence of an intramolecular chelate formed between phenolic hydroxyl and acetyl oxygen of the xanthoxyline residue. The 1:1 co-crystal of **3a** with acetonitrile (**P-I**) was the only example with one molecule of **3a** in the asymmetric unit. All other examples (**P-II** to **P-V**) revealed two independent monophyllidin molecules **A** and **B,** which differed on their part in the intramolecular hydrogen bonding mode. The interplay between zwitterionic state of proline, chelate, and different intramolecular hydrogen bonding enables (–)-monophyllidin to keep water molecules in an ordered state by multiple directional interactions.

The high ability of (–)-monophyllidin to host water in an ordered state may relate to the biological functions of the molecule in Nature. It may function as a water-preserving reservoir at elevated environmental temperatures or may protect from ice crystal formation at low temperatures. It might also be possible that (–)-monophyllidin serves as a shuttle for water molecules within plants. Since it is known that (–)-monophyllidin shows antibacterial activity, it is also likely that this molecule serves to protect plants from a distinct microorganism or an herbivore, as many other secondary metabolites do. Widespread abundance of (–)-monophyllidin is to be expected since the precursor compound, xanthoxyline, was isolated from many sources. Therefore, it would not be surprising if (–)-monophyllidin would show one of its many faces after being isolated from another plant source.

## 4. Materials and Methods

### Chemistry

#### General Information

**Starting materials** were purchased from various commercial sources and were used without further purification. (*R*)-Proline (858919-5G) and (*S*)-proline (W331902-100G-K) were purchased from Sigma-Aldrich (Sigma Aldrich, St. Louis, MO, USA). Solvents used in the synthesis and chromatographic purification steps were distilled prior to use (ethyl acetate, *n*-hexane).

**Reaction monitoring** was performed by thin-layer chromatography (TLC) on Merck silica gel 60-F254 glass plates or on Macherey & Nagel (Düren, Germany) POLYGRAM SIL G/UV 254 aluminum foils. The plates were developed with mixtures of *n*-hexane/ethyl acetate, neat ethyl acetate and ethyl acetate/MeOH/aq. ammonia. Compound spots were visualized by UV (254 nm) irradiation in a dual lamp UV cabinet (CAMAG, Muttenz, Switzerland) or in a chamber containing iodine adsorbed on silica gel.

**Purification of compounds** was performed by middle-pressure liquid chromatography (MPLC) on silica gel 60 from (Merck, 0.040–0.063 μm, 240–400 mesh, Kenilworth, NJ, USA). Stationary phase material and MPLC system consisting of unique home-built columns, an FMI pump (Fluid Metering, Inc., Syosset, Nassau County, NY, USA) and an Amersham Superfrac fraction collector was provided by MoleculeCrafting HuGs e.U., FN439498x, Vienna, Austria from private ownership.

Melting points (mp) were determined with a Bausch & Lomb microscope (Rochester, NY, USA) equipped with a Kofler melting stage and are uncorrected. 

LCMS was performed with a Waters Autopurification system. Mass spectra were taken with an ACQUITY QDa Detector (Waters Corporation, Milford, MA, USA). ESI^+^ and ESI^−^ ionization were used.

**NMR** spectra were recorded at the NMR Center of the Faculty of Chemistry, University of Vienna, on Bruker instruments (Bruker BioSpin, Rheinstetten, Germany) either on a DRX 400 WB spectrometer (resonance frequencies 400.13 MHz for ^1^H, 100.61 MHz for ^13^C), or on an Avance III HDX 700 spectrometer equipped with a quadruple QCIF cryoprobe (700.40 MHz for ^1^H, 176.12 MHz for ^13^C). The software used for processing of 1D- (^1^H, ^13^C APT or DEPTq) and 2D- (COSY, HMBC, HSQC) NMR spectra was SpinWorks 3.1.7 (copyright 2010, Kirk Marat, University of Manitoba). Coupling constants (*J*) are given in Hertz (Hz) and refer to the first-order interpretation (apparent coupling constants *J*_app_ are given). The solvent used for NMR spectroscopy: CDCl_3_, chloroform-d_1_ (CAS RN 865-49-6), was filtered through basic, activated aluminum oxide (Sigma Aldrich, St. Louis, MO, USA) prior to use. 2D NMR techniques used for the assignment of ^1^H and ^13^C resonance signals: HSQC (heteronuclear single quantum coherence), HMBC (heteronuclear multiple bond correlation), and COSY (correlation spectroscopy). Chemical shift calibration [7]: CDCl_3_, ^1^H residual solvent signal δ = 7.26, ^13^C solvent signal δ = 77.16; DMSO_6_, ^1^H residual solvent signal δ = 2.50, ^13^C solvent signal δ = 39.52.

**Optical rotation** was measured on a Perkin Elmer Polarimeter 341 (Waltham, MA, USA), with a 100 mm path length cell, in combination with a Julabo 5 thermostat. The measured temperature is stated in all cases. The wavelength of the light used was 589 nm (sodium D line) in all cases.

ee-Determinations with SFC were carried out on an Agilent system equipped with an Aurora-Box (G4301A), a binary pump (G4302A), an autosampler (G1313A), a DAD-detector (G1315C) and an MSD (mass selective detector) (G6140A). Chromatographic separation was obtained by isocratic elution (MeOH containing 0.1% diethyl amine/CO_2_ = 40/60) using a Phenomenex Lux Cellulose 4 column (150 × 2 mm; 5 µm) at room temperature. The enantiomers were detected at 230 nm. The injection volume was 5 µL, the flow 1.2 mL/min, and the back pressure was set to 150 bar. The sample concentration was 1 mg/mL.

X-ray intensity data were measured on Bruker X8 APEX2 and D8-Venture diffractometer equipped with multilayer monochromators, Mo K-α INCOATEC microfocus sealed tubes and Kryoflex and Oxford cooling devices. The structures were solved by direct methods and refined by full-matrix least-squares techniques. Non-hydrogen atoms were refined with anisotropic displacement parameters. Hydrogen atoms were inserted at calculated positions and refined with a riding model or as rotating groups. The following software was used: Frame integration, *Bruker SAINT software package* (v7.68A, v8.38A © 2020-2019 Bruker AXS) using a narrow-frame algorithm, Absorption correction, *SADABS* [8], structure solution, *SHELXS-2013* [9], refinement, *SHELXL-2013* [9], *Olex2* [10], *SHELXLE* [11], molecular diagrams, *Olex2* [10]. Experimental data and CCDC-code can be found in Table S1 (see SM). Crystal data, data collection parameters, and structure refinement details are given in Tables S2–S15 (see SM). Supplementary crystallographic data can be obtained free of charge via http://www.ccdc.cam.ac.uk/conts/retrieving.html from the CCDC.

#### Synthetic procedures (for NMR interpretation and spectra see Supplementary Materials)

● 2-hydroxy-4,6-dimethoxyacetophenone (**1**) (CAS RN 90-24-4)

Xanthoxyline (**1**) was prepared as reported [12] on a 1 gram scale affording 0.542 g of **1** (42% yield).

● (–)-Monophyllidin (**3a**) (CAS RN 1345098-22-7)

A mixture of 2-hydroxy-4,6-dimethoxyacetophenone (**1**, 0.7 g, 3.57 mmol), formaldehyde (37%, 0.32 mL, 4.3 mmol) and (*S*)-proline (**2a**, 0.493 g, 4.28 mmol) in ethanol (4 mL) was prepared in a screw cap tube. The tube was sealed and kept in an oil bath at 80 °C for 15 h. The solvent was removed under reduced pressure, and the residue was taken up with water. Upon setting pH to 10 by addition of aqueous ammonia, unreacted starting material rapidly precipitated and was filtered off. The aqueous solution was adjusted with NH_4_Cl to pH 9 and extracted four times, with 25 mL of EtOH/CHCl_3_ = 1/4. The solvents of the combined organic phases were removed under reduced pressure.

The resulting residue was purified by column chromatography (silica gel, eluent: ethyl acetate: MeOH = 3:1) to give (*S*)-1-(3-acetyl-2-hydroxy-4,6-dimethoxybenzyl)-pyrrolidine-2-carboxylic acid (**3a**, 0.737 g, 2.28 mmol, 64% yield, mp: 157–158 °C (dec.). LCMS: [M-H]^−^ calculated for C_16_H_20_NO_6_: 322.34, found: 322.27. Optical rotation: ${\left[\alpha \right]}_{20}^{D}$ = −12.5°, [c = 1.00, CHCl_3_]; −8° [c = 0.28, CHCl_3_]. Enantiomeric purity: ee > 98%, t_R_ = 2.4 min (for chromatograms see Supplementary Materials).

● (+)-Monophyllidin (**3b**)

A mixture of 2-hydroxy-4,6-dimethoxyacetophenone (**1**) (0.344 g, 1.752 mmol), formaldehyde (37%, 0.156 mL, 2.102 mmol) and (*R*)-proline (**2b**) (0.242 g, 2.102 mmol) in ethanol (4 mL) was prepared in a screw cap tube. The tube was sealed and kept in an oil bath at 80 °C for 15 h. The solvent was removed under reduced-pressure and the residue was taken up with water. Upon setting pH to 10 by addition of aqueous ammonia unreacted starting material precipitated in a rapid manner and was filtered off. The aqueous solution was acidified with NH_4_Cl to pH 9 and four times extracted with 25 mL of EtOH/CHCl_3_ = 1/4. The solvents of the combined organic phases were removed under reduced pressure.

The resulting residue was purified by column chromatography (silica gel, eluent: ethyl acetate: MeOH = 3:1). (*R*)-1-(3-acetyl-2-hydroxy-4,6-dimethoxy-benzyl)-pyrrolidine-2-carboxylic acid (**3b**, 0.142 g, 0.44 mmol, 25% yield, mp: 148 °C (dec); optical rotation: ${\left[\alpha \right]}_{20}^{D}$ = +11.2°, (c = 1.00, CHCl_3_); enantiomeric purity: ee = 93%, t_R_ = 3.3 min (for ee-determination see Supplementary Materials).

● (*S*)-(1*S*,2*R*,5*S*)-2-isopropyl-5-methylcyclohexyl pyrrolidine-2-carboxylate (**4**)

A mixture of (*S*)-proline (**2a**) (5.76 g, 50 mmol), (1*S*,2*R*,5*S*)-(+)-menthol (12.03 g, 77 mmol), *p*-toluene sulfonic acid monohydrate (12.00 g, 63.1 mmol) was introduced in 100 mL of toluene. The heterogeneous mixture obtained was refluxed in a Dean-Stark apparatus for 16 h. The nascent water (ca. 2 mL) was removed azeotropically. The insoluble materials were removed by filtration. The toluene solution was concentrated to ca. 30 mL at reduced pressure and diluted with 30 mL of Et_2_O. Subsequently, the organic phase was extracted with saturated sodium hydrogen carbonate solution, washed once with water and dried over sodium sulfate. The solution was concentrated *in vacuo*. To the residual material, 20 mL of hydrogen chloride (4M in dioxane) was added. Crystallization of **4xHCl** was induced by addition of *n*-hexane and cooling to 0°C. The crystals were filtered and washed with dry ethyl ether and petroleum ether. Two crops afforded 7.385 g (51% yield) of **4xHCl** as colorless crystals. 1 g of the hydrochloride was neutralized with 2M NaOH to afford 0.856 g (98% yield) of the free base **4** as a colorless oil. LCMS: [M+H]^+^: calculated for C_15_H_27_NO_2_: 254.21, found: 254.29. Optical rotation: ${\left[\alpha \right]}_{20}^{D}$ = +36.2°, (c = 1.00, MeOH).

● (*S*)-(1*S*,2*R*,5*S*)-2-isopropyl-5-methylcyclohexyl-1-(3-acetyl-2-hydroxy-4,6-dimethoxybenzyl)- pyrrolidine-2-carboxylate (**5**)

A mixture of 2-hydroxy-4,6-dimethoxyacetophenone (**1**) (0.294 g, 1.50 mmol), formaldehyde (37%, 0.13 mL, 1,80 mmol) and the hydrochloride of (*S*)-(1*S*,2*R*,5*S*)-2-isopropyl-5-methylcyclohexyl pyrrolidine-2-carboxylate (**4xHCl**) (0,521 g, 1,798 mmol) in ethanol (4 mL) was prepared in a screw cap tube. The tube was sealed and kept in an oil bath at 90 °C for 48 h. The mixture obtained was neutralized with aq. NaHCO_3_ and extracted with ethyl acetate. The solvent was removed under reduced pressure on a rotary evaporator. The resulting residue was purified by column chromatography (*n*-hexane/ethyl acetate) to give (*S*)-(1*S*,2*R*,5*S*)-2-isopropyl-5-methylcyclohexyl 1-(3-acetyl-2-hydroxy-4,6-dimethoxybenzyl)-pyrrolidine-2-carboxylate (**5**) (0.373 g, 0.808 mmol, 54% yield). LCMS: [M + H]^+^: calculated for C_26_H_39_NO_6_: 426.29, found: 462.46; mp: 96 °C.

● Hydrochloride of (–)-monophyllidin-(+)-menthyl ester (**5xHCl**)

(2*S*)-1-(3-acetyl-2-hydroxy-4,6-dimethoxybenzyl)-2-(((1*S*,2*R*,5*S*)-2-isopropyl-5-methylcyclohexyloxy) carbonyl) pyrrolidinium chloride (**5xHCl**) was synthesized by treating a solution of **5** in MeOH with hydrogen chloride (4M) in dioxane (mp: 171–172 °C).

## Figures and Tables

**Table 1:** Summary of (–)-monophyllidine (**3a**) polymorphs obtained from different solvents.

Solvent	ID	Space Group	mp [°C]	Crystal Habitus
acetonitrile	P-I	*P2_1_2_1_2_1_*	161–162	block-like
acetonitrile	P-II	*C2*	158–160	needles
acetonitrile	P-VI	*P2_1_2_1_2_1_*	nd (singleton)	plates
chloroform	P-III	*P1*	158–159	needles
ethanol-acetonitrile	P-IV	*P1*	158–159	needles
water	P-V	*P2_1_*	155–156	blocks

**Table 2:** Summary of (–)-monophyllidin polymorphs **P-I** to **P-VI**.

ID	Space Group	mp [°C]	Crystal Habitus	Guest Molecules	(–)-3a: Guest(s)
P-I	*P2_1_2_1_2_1_*	161–162	blocks	CH_3_CN	1:1
P-II	*C2*	158–160	needles	CH_3_CN:water	2:1:2
P-III	*P1*	158–159	needles	CHCl_3_:water	2:2:1
P-IV	*P1*	158–159	needles	H_2_O	2:5
P-V	*P2_1_*	155–156	blocks	H_2_O	2:1
P-VI	*P2_1_2_1_2_1_*	nd	plates	H_2_O	2:1

## Supplementary Materials

The following are available online at https://www.mdpi.com/1420-3049/25/3/449/s1, Supplementary Materials comprise interpretation and copies of ^1^H and ^13^C NMR spectra for all compounds described, IR-spectrum of **3a**, determination of enantiomeric purity of **3a**, and **3b**, X-ray data collection and structure refinement of 3a-polymorphs **P-I** to **P-VI**, and **5xHCl** including hydrogen bond geometries, respectively.
