# Supplementary material for: A Many-Faced Alkaloid: Polymorphism of (–)-Monophyllidin
Source: Molecules. 2020 Jan 21;25(3):449. doi: 10.3390/molecules25030449 (PMC7037440; doi:10.3390/molecules25030449)
Supplement: Supplementary file 1 [file molecules-25-00449-s001.pdf]

molecules MDPI

Supporting Materials

Content:

S1. List of Compounds

S2. NMR data of synthesized compounds

S2.1 Assignment of the $^{1}H/^{13}C$ resonances of compounds

S2.2 Graphical representations of NMR spectra

S3. IR-spectrum of (–)-monophyllidin **3a**

S4. Determination of enantiomeric purity of (–)-monophyllidin **3a** and (+)-monophyllidin **3b**

S5. Crystallographic investigations

S5.1 Experimental parameter, CCDC-Codes (Table S1), sample and crystal data, data collection and structure refinement (Tables S2-S15)

S5.2 Hydrogen bond geometries for (–)-monophyllidin polymorphs **P-I** to **P-V** (Tables S16-S22)

S1. List of compounds

(1) Xanthoxyline

(3a) (–)-Monophyllidin

(**3b**) (+)-Monophyllidin

(4) (+)-menthyl ester of (*S*)-proline

(5) (+)-menthyl ester of (–)-monophyllidin

(5xHCl) hydrochloride of (+)-menthyl ester of (–)-monophyllidin

S2. NMR data for synthesized compounds

Abbreviations: br, broad; multiplicity: m, multiplet; s, singlet, d, doublet; t, triplet; q, quaternary or quartet; qu, quintet; sept, septet; sext, sextet; AB, spin system; m [t, dd, q, tt, sext]. Notation in brackets describe the overall appearance of the signal pattern (e.g., m[d] represents a multiplet with the appearance like a doublet).

S2.1 Assignment of $^{1}H/^{13}C$ Resonances of Compounds

(1) 2-hydroxy-4,6-dimethoxyacetophenone (CAS RN 90-24-4; xanthoxyline)

Numbering scheme for interpretation of NMR spectra of **1**

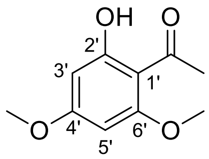

1H NMR (400.13 MHz, CDCl3, 25 °C): δ = 2.60 (s, 3H, CH3 acetyl), 3.81 (s, 3H, OCH3-4′), 3.85 (s, 3H, OCH3-6′), 5.92 (d, J = 2.4, 1H, H-5′), 6.05 (d, J = 2.4, 1H, H-3′), 14.02 (sharp s, 1H, ArOH). 13C{1H}NMR (100.61 MHz, CDCl3, 25 °C): δ = 33.04 (CH3 acetyl), 55.67 (2x OCH3), 90.87 (CH-5′), 93.62 (CH-3′), 106.14 (C-1′), 163.05 (C-6′), 166.22 (C-4′), 167.73 (C-2′), 203.29 (C=O acetyl).

(3a) (−)-Monophyllidin in $CDCl_3$

Numbering scheme for interpretation of NMR spectra of **3a**

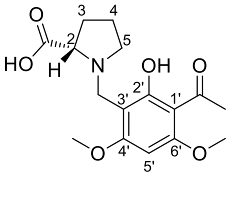

^{1}H NMR (700.40 MHz, $CDCl_3$, 25 °C): δ = 1.93-2.00 (m, 2H, $H_{a,b}$-4), 2.24-2.31 (m, 1H, H_a-3), 2.36-2.42 (m, 1H, H_b-3), 2.59 (s, 3H, C*H*3 acetyl), 2.99 (ddd, *J* = 11.2, 10.0, 7.0, 1H, H_a-5), 3.57 (ddd, *J* = 11.2, 7.0, 4.1, 1H, H_b-5), 3.83 (dd, *J* = 9.4, 3.6 Hz, 1H, *H*-2), 3.93 (s, 3H, OCH_3-4′), 3.98 (s, 3H, OCH_3-6′), 4.23 (s, 2H, CH_2 benzyl), 5.98 (s, 1H, *H*-5′), 14.52 (s, 1H, O*H*).
^{13}C{^{1}H}NMR (176.12 MHz, $CDCl_3$, 25 °C): δ = 24.20 (CH_2-4), 29.10 (CH_2-3), 33.19 (CH_3 acetyl), 46.87 (NCH_2), 54.03 (CH_2-5), 55.93 (OCH_3), 56.33 (OCH_3), 68.78 (*C*H-2), 86.17 (*C*H-5′), 98.77 (*C*-3′), 105.85 (*C*-1′), 164.59 (*C*-6′), 165.02/165.04 (*C*-2′/4′), 170.56 (*C*OOH), 203.78 (*C*=O acetyl).

(4) (*S*)-(1*S*,2*R*,5*S*)-2-isopropyl-5-methylcyclohexyl pyrrolidine-2-carboxylate

Numbering scheme for NMR interpretation of **4**

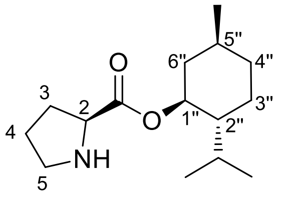

^{1}H NMR (400.13 MHz, $CDCl_3$, 25 °C): δ = 0.75 (d, ^{3}J = 7.0, 3H, CH_3 isopropyl), 0.80-0.92 (m, 1H, H_a-4′′), 0.89 (d, ^{3}J = 7.0, 3H, CH_3 isopropyl), 0.90 (d, ^{3}J = 6.4, 3H, CH_3-5′′), 0.97 (ddd, *J* = 12.1, 12.1, 11.0, 1H, H_a-6′′), 0.98-1.11 (m, 1H, H_a-3′′), 1.40 (dddd, ^{3}J = 12.3, 10.9, 3.1, 2.9, 1H, *H*-2′′), 1.43-1.54 (m, 1H, *H*-5′′), 1.63-1.71 (m, 1H, H_b-3′′), 1.65-1.71 (m, 1H, H_b-4′′), 1.69-1.81 (m, 2H, CH_2-4), 1.75-1.85 (m, 1H, H_a-3), 1.82-1.91 (m, 1H, C*H* isopropyl), 1.96-2.02 (m, 1H, H_b-6′′), 2.06 (br s, 1H, N*H*), 2.08-2.18 (m, 1H, H_b-3), 2.88 (ddd, ^{3}J = 10.2, 6.9, 6.3, 1H, H_a-5), 3.05-3.12 (m, 1H, H_b-5), 3.70 (dd, ^{3}J = 8.7, 5.8, 1H, *H*-2), 4.70 (ddd, ^{3}J = 10.9, 10.9, 4.4, 1H, *H*-1′′).
^{13}C{^{1}H}NMR (100.61 MHz, $CDCl_3$, 25 °C): δ = 16.19 (CH_3 isopropyl), 20.94 (CH_3 isopropyl), 22.13 (CH_3-5′′), 23.38 (CH_2-3′′), 25.66 (CH_2-4), 26.35 (CH isopropyl), 30.64 (CH_2-3), 31.53 (CH-5′′), 34.37 (CH_2-4′′), 40.97 (CH_2-6′′), 47.14 (CH_2-5), 47.16 (CH-2′′), 60.30 (CH-2), 74.98 (CH-1′′), 175.19 (*C*=O ester).

(5) (*S*)-(1*S*,2*R*,5*S*)-2-isopropyl-5-methylcyclohexyl-1-(3-acetyl-2-hydroxy-4,6-dimethoxy-benzyl pyrrolidine-2-carboxylate

Numbering scheme for NMR interpretation of **5**:

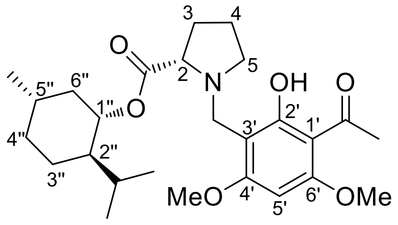

^{1}H NMR (700.40 MHz, $CDCl_3$, 25 °C): δ = 0.72 (d, ^{3}J = 7.0, 3H, CH_{3a}-isopropyl), 0.82-0.88 (m, 4H, CH_a-4′′, CH_{3b}-isopropyl), 0.90 (d, ^{3}J = 7.0, 3H, CH_3-5′′), 0.97 (ddd, *J* = 11.6, 11.6, 11.6, 1H, H_a-6′′), 1.02 (m[ddd], 1H, CH_a-3′′), 1.41 (m[ttt],1H, C*H*-2′′), 1.48 (m,1H, C*H*-5′′), 1.61-1.69 (m, 3H, CH_b-3′′, CH_a-4, CH_b-4′′), 1.77-1.87 (m, 2H, H_a-3, CH_b-4, 1.93 (dddd, *J* = 13.9, 6.9, 6.9, 2,8, 1H, C*H* isopropyl), 1.97 (m, 1H, H_b-6′′), 2.02-2.04 (m, 1H, H_b-3), 2.38 (m[dd], 1H, H_a-5), 2.59 (s, 3H, CH_3 acetyl), 3.10 (m[t], 1H, H_b-5), 3.14 (m[t], 1H, *H*-2), 3.85 (s, 3H, OCH_3), 3.89 (s, 3H, (OCH_3), 3.91 (AB-system, J_{AB} = -13.3, 2H, CH_2 benzyl), 4.71 (ddd, *J* = 10.9, 10.9, 4.3, 1H, C*H*-1′′), 5.93 (s, 1H, *H*-5′), 13.5-14.3 (br, 1H, ArO*H*). ^{13}C{1H}NMR (175 MHz, $CDCl_3$, 23 °C): δ = 15.95 (CH_{3a}-isopropyl), 20.96 (CH_{3b}-isopropyl), 22.18 (CH_3-5′′), 22.52 (CH_2-4), 23.18 (CH_2-3′′), 26.04 (CH-isopropyl), 29.86 (CH_2-3), 31.47 (CH-5′′), 33.28 (CH_3 acetyl), 34.39 (CH_2-4′′), 40.89 (CH_2-6′′), 43.00 (CH_2 benzyl), 46.92 (CH-2′′), 52.83 (CH_2-5), 55.49 (OCH_3), 55.62 (OCH_3), 64.01 (CH-2), 73.98 (CH-1′′), 85.63 (CH-5′), 105.11 (*C*-3′), 106.11 (*C*-1′), 162.72 (*C*-6′), 164.60 (*C*-2′), 165.00 (*C*-4′), 173.97 (*C*=O ester), 203.25 (*C*=O acetyl).

(5xHCl) Hydrogen chloride of (+)-menthyl ester of (–)-monophyllidin

Numbering scheme for NMR interpretation of **5xHCl**:

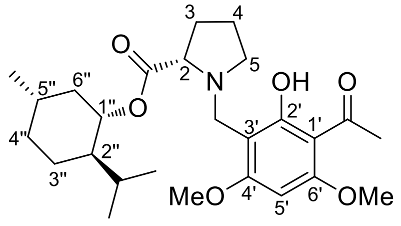

x HCl

^{1}H NMR (700.40 MHz, DMSO-d$_6$, 25 °C): δ = 0.69 (d, ^{3}J = 7.0, 3H, C$H3_a$-isopropyl), 0.82-0.86 (m, 4H, CH_{3b}-isopropyl, H_a-4′′), 0.89 (d, ^{3}J = 7.0, 3H, CH_3-5′′), 0.94 (m[ddd], $|^{2}J_{AB}|$ = $^{3}J_1$ ax,ax = $^{3}J_2$ ax,ax = 11.8, 1H, H_a-6′′), 1.03 (m, 1H, H_a-3′′), 1.39 (dddd[tt], *J* = 11.6, 11.6, 2.9, 2.9, 1H, *H*-2′′), 1.46 (m, 1H, *H*-5′′), 1.60-1.67 (m, 2H, H_a-4′′, H_b-3′′), 1.73-1.79 (m, 2H, C*H*-isopropyl, H_b-6′′), 1.82 (m, 1H, H_a-3), 1.93 (m, 1H, H_a-4), 1.98 (m, 1H, H_b-4), 2.44 (m, 1H, H_b-3), 2.59 (s, 3H, COCH_3), 3.11 (1H, H_a-5), 3.54 (1H, H_b-5), 3.98 (s, 3H, OCH_3), 4.00 (s, 3H, OCH_3), 4.20 (m[t], br, *H*-2), 4.29 (AB, br, $^{2}J_{AB}$ = -13.4, NCH_2), 4.63 (ddd, ^{3}J = 10.9, 10.9, 4.4, 1H, *H*-1′′), 6.35 (s, 1H, *H*-5′), 10.23 (br, 1H, N^{+}*H*), 14.67 (s, 1H, C2′-O*H*); ^{13}C{^{1}H}NMR (176.12 MHz, DMSO-d$_6$, 25 °C): δ = 15.83 (CH_3-isopropyl), 20.50 (CH_3-isopropyl), 21.33 (*C*-4), 21.84 (CH_3-5′′), 22.60 (*C*-3′′), 25.71 (*C*H-isopropyl), 27.96 (*C*-3), 30.65 (*C*-5′′), 32.86 ($COCH_3$), 33.48 (*C*-4′′), 40.02 (*C*-6′′), 44.43 (NCH_2), 46.06 (*C*-2′′), 53.72 (*C*-5), 56.57 (OCH_3), 56.71 (OCH_3), 64.19 (*C*-2), 75.93 (*C*-1′′), 87.78 (*C*-5′), 97.03 (C_q-3′), 104.88 (C_q-1′), 164.87 (*C*-2′, via HMBC of 2′-O*H*), 165.03 (*C*-4′), 165.29 (*C*-6′), 167.92 (COO-menthyl ester), 203.59 ($COCH_3$).

S2.2 Representation of ^{1}H/^{13}C NMR spectra of compounds

(3a) (–)-Monophyllidin in $CDCl_3$:

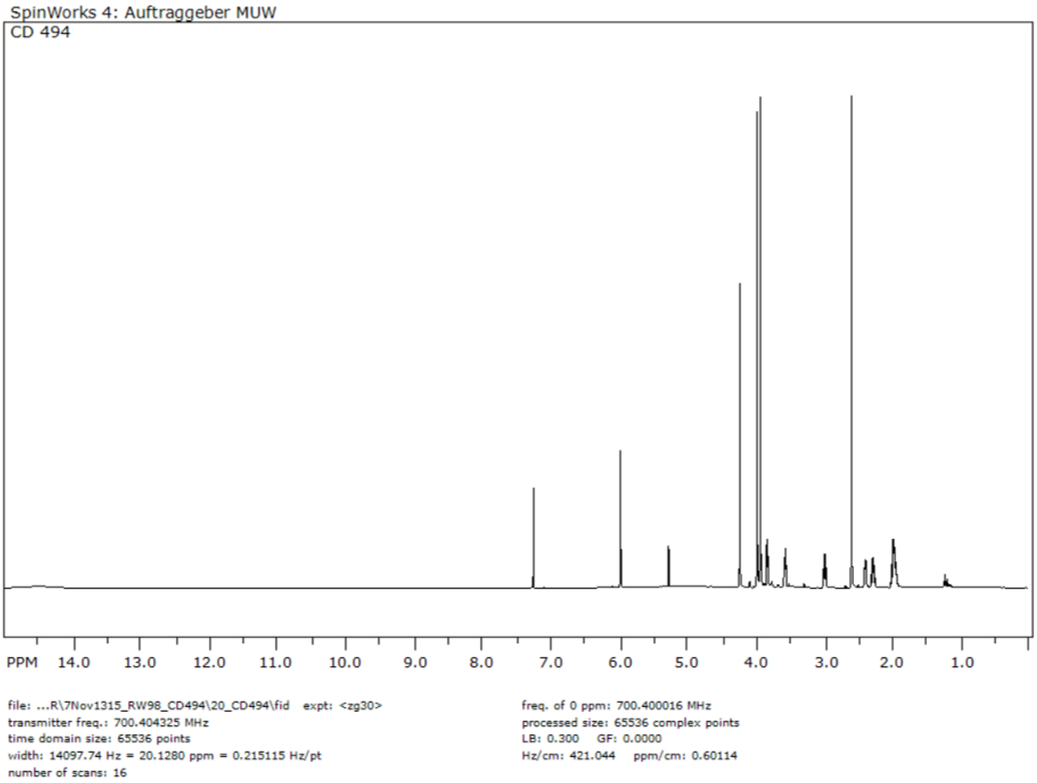

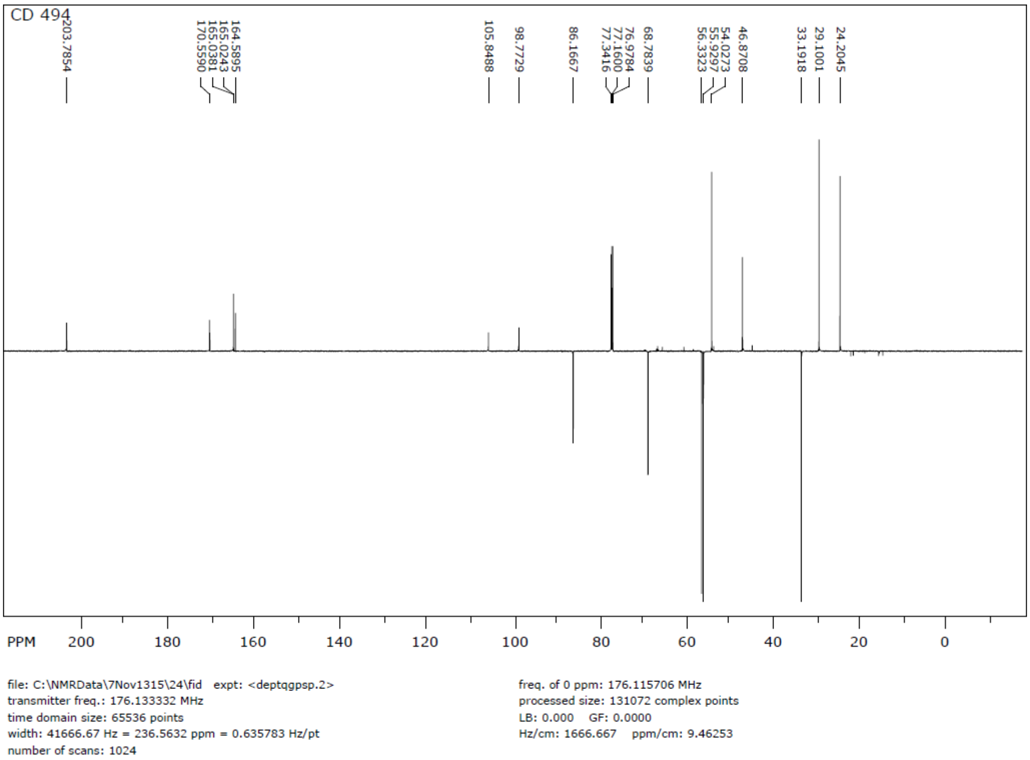

(3b) (+)-Monophyllidin in $CDCl_3$:

Overlay of ^{1}H-NMR of (+)-monophyllidin 3b (lower trace) with (–)-monophyllidin **3a** (upper trace) (zoom 1.0-6.2 ppm):

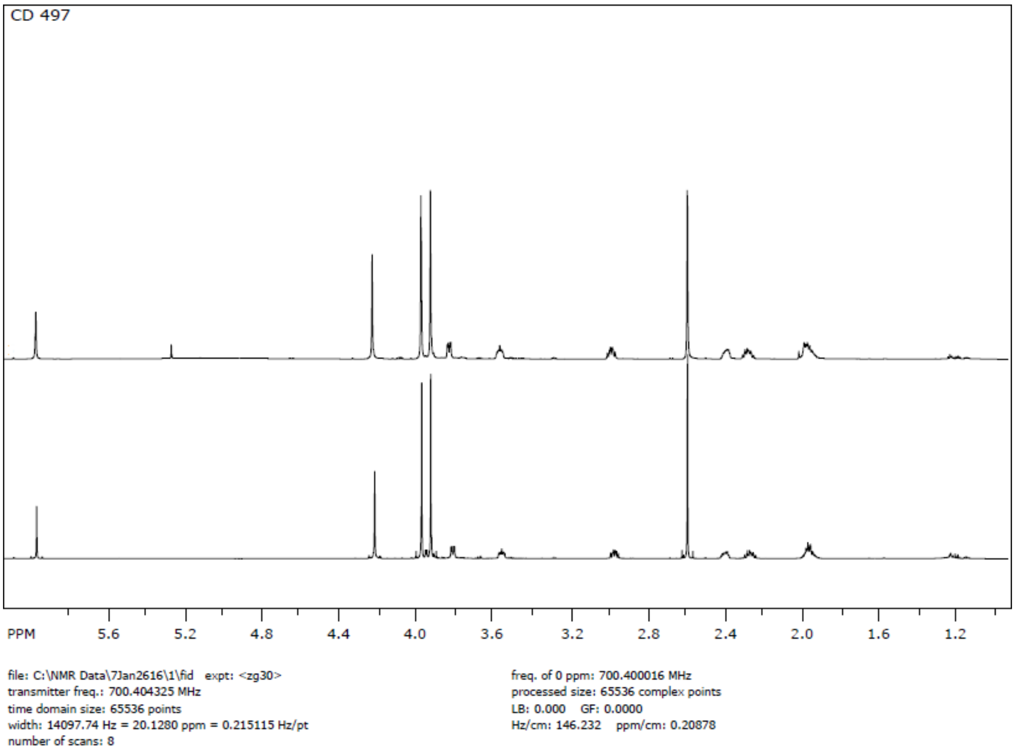

(4) (*S*)-(1*S*,2*R*,5*S*)-2-isopropyl-5-methylcyclohexyl pyrrolidine-2-carboxylate

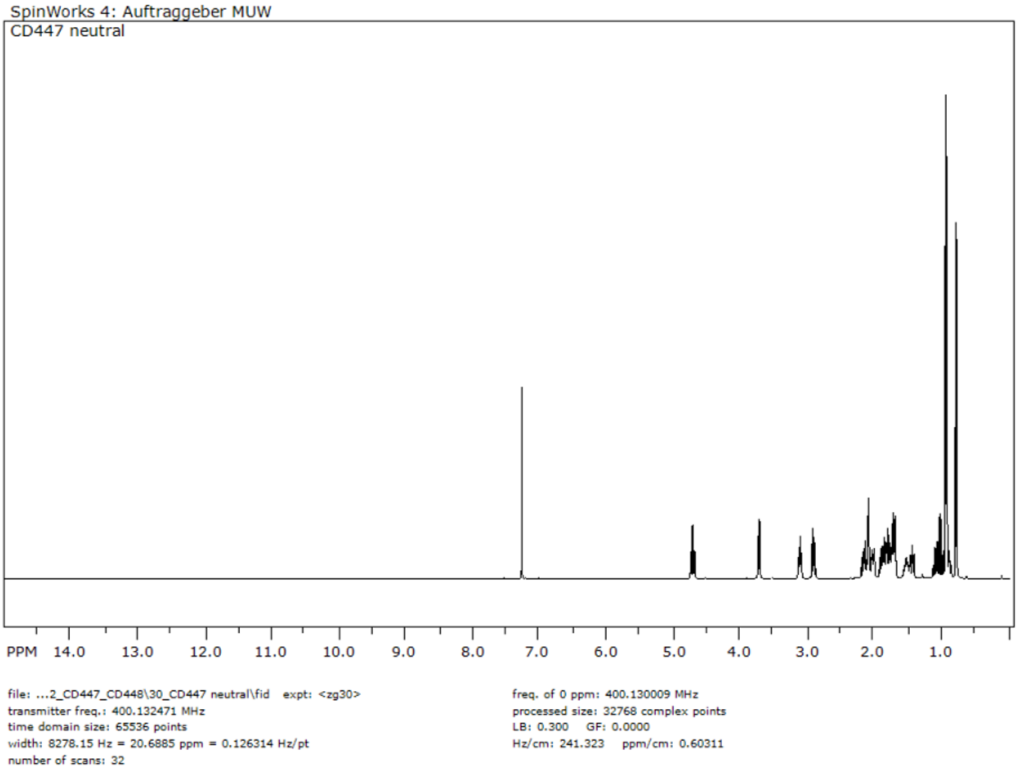

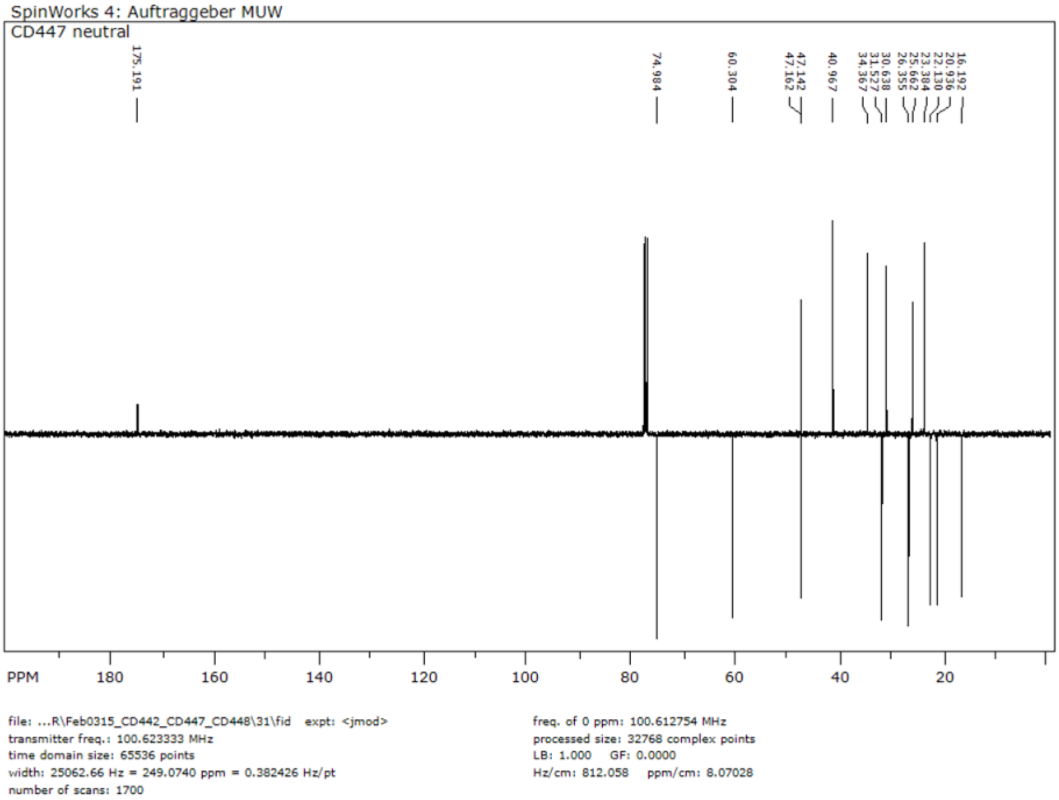

(5) (*S*)-(1*S*,2*R*,5*S*)-2-isopropyl-5-methylcyclohexyl-1-(3-acetyl-2-hydroxy-4,6-dimethoxy-benzyl)-pyrrolidine-2-carboxylate

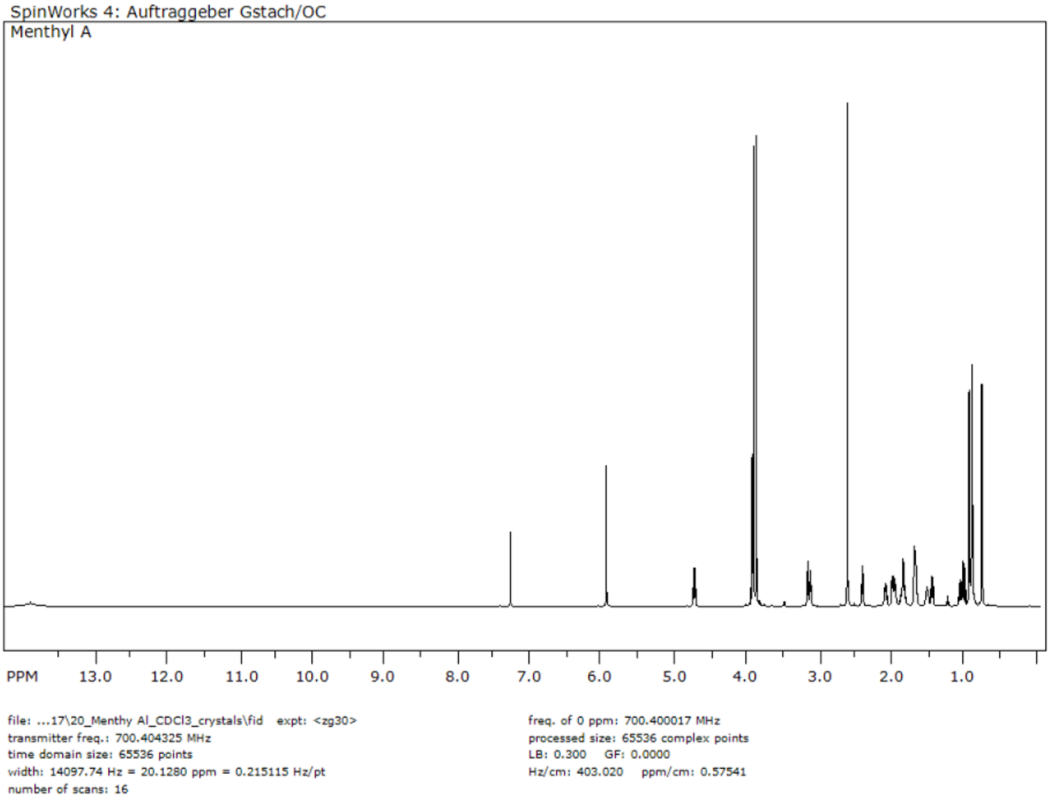

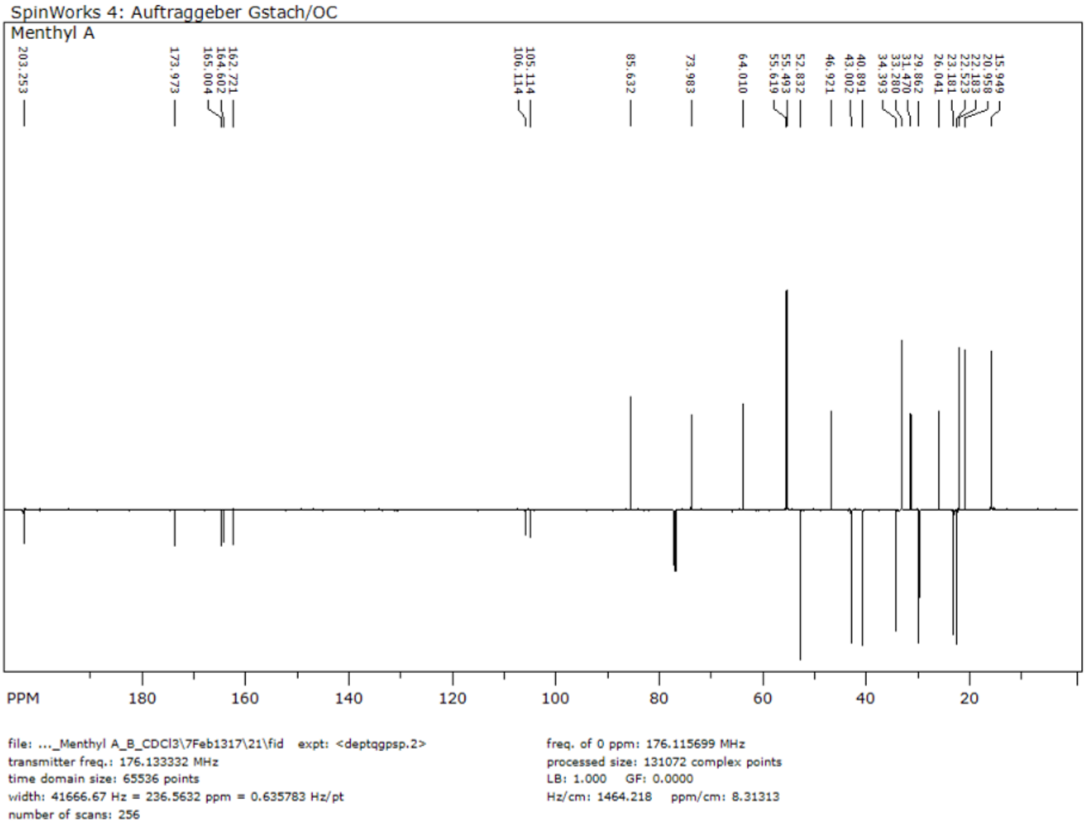

(5xHCl) Hydrochloride of (+)-menthyl ester of (–)-monophyllidin 5

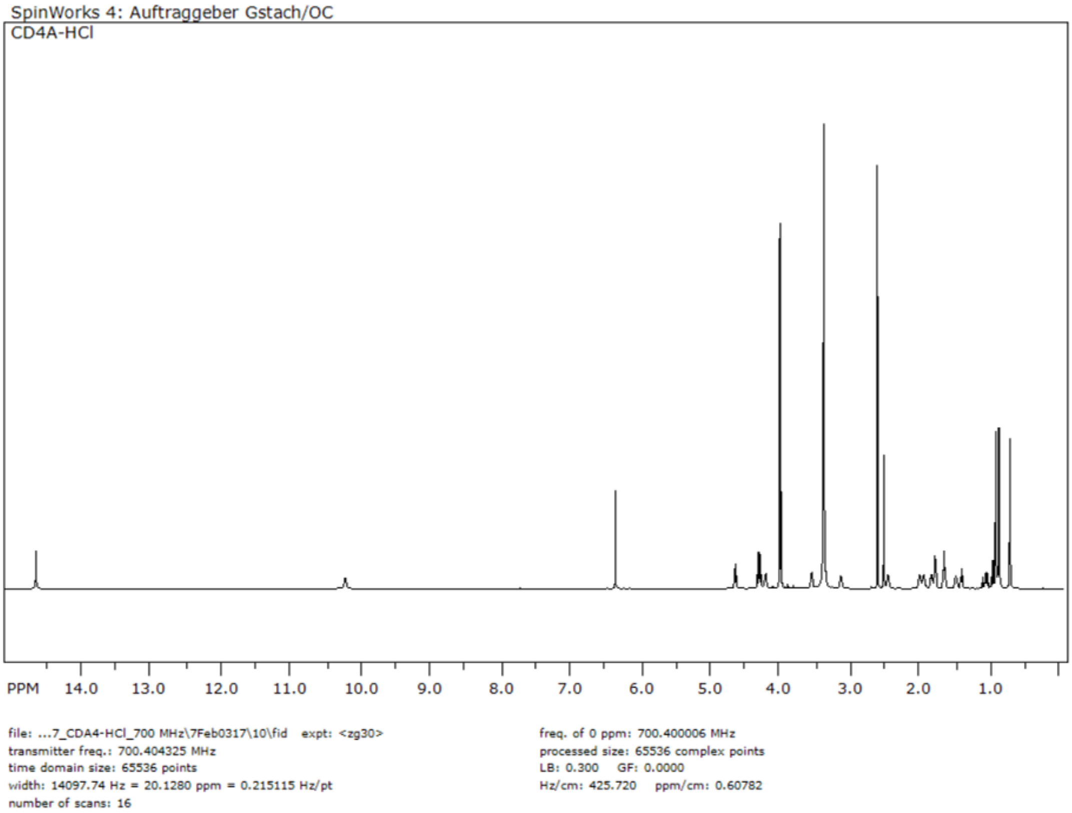

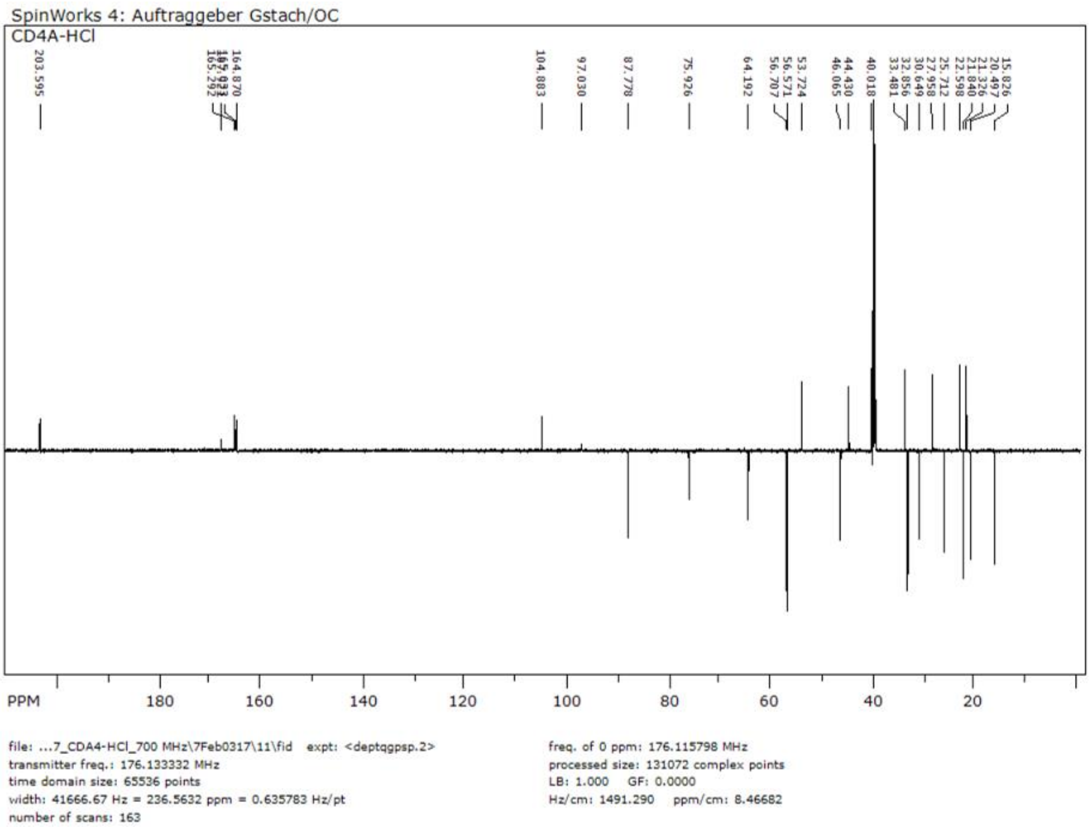

S3. IR-Spectrum of synthesized (–)-monophyllidin 3a.

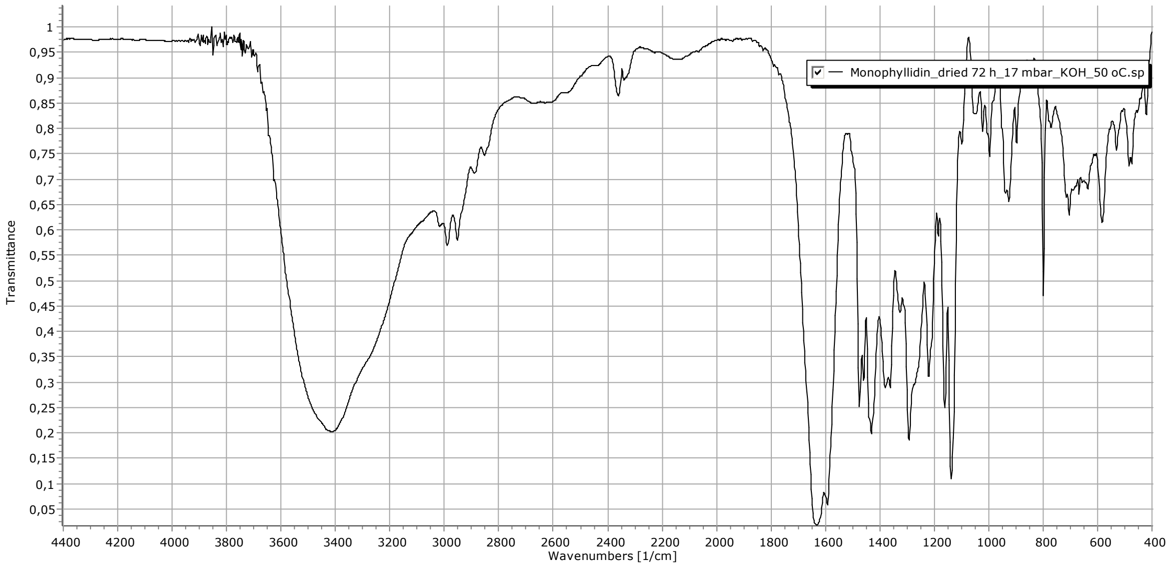

Figure S1. IR-spectrum of synthesized (–)-monophyllidin **3a** (1 mg; KBr-pellet; dried over KOH at 50 °C, 17 mbar for 72 h).

S4. Determination of enantiomeric purity of synthesized 3a and 3b

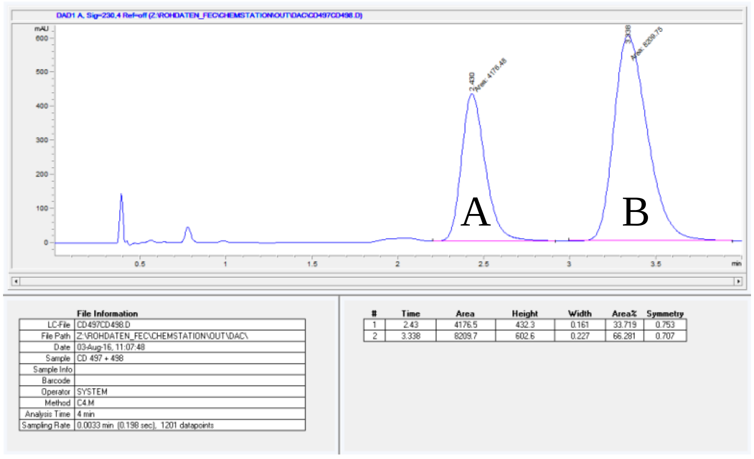

Figure S2. Mixture of (–)-monophyllidin (**3a**) (peak A) and (+)-monophyllidin (**3b**) (peak B)

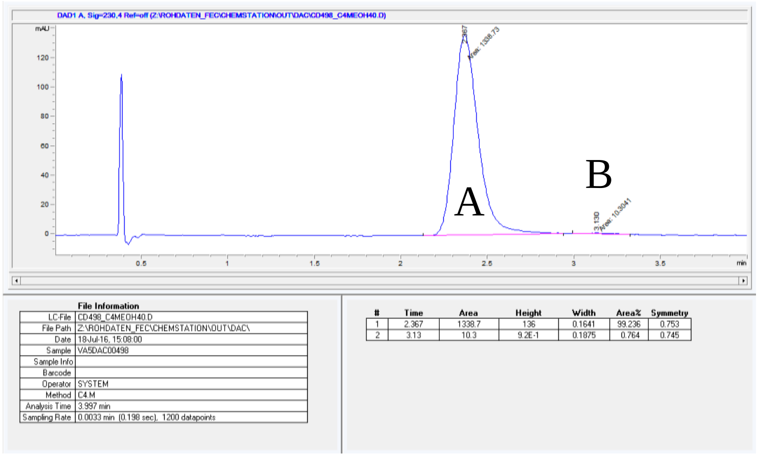

Figure S3. Synthesized (–)-monophyllidin (**3a**): ee > 98 %, t_R = 2.4 min (peak A).

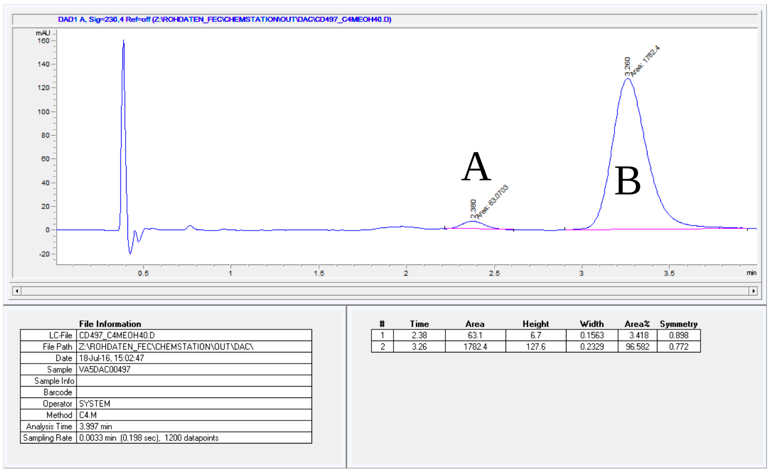

Figure S4. Synthesized (+)-monophyllidin (**3b**): ee = 93%, t_R = 3.3 min (peak B).

S5 Crystallographic investigations

S5.1 Experimental parameter, CCDC-codes sample and crystal data, data collection and structure refinement of (–)-monophyllidin polymorphs

Table S1. Experimental parameters and CCDC-Code**s**.

Sample	Machine	Source	Temp.	Detector Distance	Time/ Frame	#Frames	Frame width	CCDC
	Bruker		[K]	[mm]	[s]		[°]	
P-I	D8	Mo	100	40	1	600	0.3	1973088
P-II	D8	Mo	100	40	8	2114	0.5	1973089
P-III	D8	Mo	100	34	40	1824	0.8	1973084
P-IV	D8	Mo	100	40	30	1266	0.8	1973086
P-V	D8	Mo	100	40	5	1091	1.0	1973085
P-VI	D8	Mo	100	40	140	490	0.5	1973087
5xHCl	X8	Mo	130	55	10	1068	0.5	1500946

Table S2. Sample and crystal data of (–)-monophyllidin polymorph **P-I**.

Chemical formula	$C_{18}H_{24}N_2O_6$	Crystal system	orthorhombic	
Formula weight [g/mol]	364.39	Space group	*P212121*	
Temperature [K]	130	Z	4	
Measurement method	\Φ and \ω scans	Volume [Å^3]	1766.8(4)	
Radiation (Wavelength [Å])	MoKα (λ = 0.71073)	Unit cell dimensions [Å] and [°]	7.0524(10)	90
Crystal size / [mm^3]	0.1 × 0.09 × 0.05		8.5308(7)	90
Crystal habit	clear colourless block-like		29.366(4)	90
Density (calculated) / [g/cm^3]	1.37	Absorption coefficient / [mm^{-1}]	0.103	
Abs. correction Tmin	0.6012	Abs. correction Tmax	0.7452	
Abs. correction type	multi-scan	F(000) [e$^-$]	776	

Table S3. Data collection and structure refinement of (–)-monophyllidin polymorph **P-I**.

Index ranges	-5 ≤ h ≤ 8, -6 ≤ k ≤ 10, -35 ≤ l ≤ 35	Theta range for data collection [°]	2.774 to 50.69	
Reflections number	6721	Data / restraints / parameters	3223/1/238	
Refinement method	Least squares	Final R indices	all data	R1 = 0.1356, wR2 = 0.1385

Function minimized	Σ w(F_o^2 - F_c^2)^2		I>2σ(I)	R1 = 0.0639, wR2 = 0.1079
Goodness-of-fit on F2	1.012	Weighting scheme	w=1/[σ^2(Fo^2)+(0.0380P)^2+0.9359P]	
Largest diff. peak and hole [e Å-3]	0.31/-0.27		where P=(Fo^2+2Fc^2)/3	

Table S2. Sample and crystal data of [**P-II**]

Chemical formula	$C_{17}H_{24.5}N_{1.5}O_7$	Crystal system	monoclinic	
Formula weight [g/mol]	361.88	Space group	*C2*	
Temperature [K]	100	Z	8	
Measurement method	\f and \w scans	Volume [Å^3]	3466.1(6)	
Radiation (Wavelength [Å])	MoK*α* (*λ* = 0.71073)	Unit cell dimensions [Å] and [°]	22.879(2)	90
Crystal size / [mm^3]	0.341 × 0.147 × 0.068		7.2921(8)	92.795(6)
Crystal habit	clear colourless needle		20.800(2)	90
Density (calculated) / [g/cm^3]	1.387	Absorption coefficient / [mm^{-1}]	0.108	
Abs. correction Tmin	0.6876	Abs. correction Tmax	0.746	
Abs. correction type	multiscan	F(000) [e$^-$]	1544	

Table S3. Data collection and structure refinement of [**P-II**]

Index ranges	-31 ≤ h ≤ 32, -10 ≤ k ≤ 10, -29 ≤ l ≤ 29	Theta range for data collection [°]	5.17 to 60.062	
Reflections number	63934	Data / restraints / parameters	10153/1/479	
Refinement method	Least squares	Final R indices	all data	R1 = 0.0552, wR2 = 0.0963
Function minimized	Σ w(F_o^2 - F_c^2)^2		I>2σ(I)	R1 = 0.0424, wR2 = 0.0871
Goodness-of-fit on F2	1.03	Weighting scheme	w=1/[σ^2(Fo^2)+(0.0321P)^2 +3.1739P]	
Largest diff. peak and hole [e Å-3]	0.38/-0.22		where P=(Fo^2+2Fc^2)/3	

Table S6. Sample and crystal data of **P-III**.

Chemical formula	$C_{17}H_{23}Cl_3NO_{6.5}$	Crystal system	triclinic
Formula weight [g/mol]	451.71	Space group	*P1*
Temperature [K]	100	Z	2

Measurement method	\f and \w scans	Volume [Å^3]	997.26(7)	
Radiation (Wavelength [Å])	MoKα (λ = 0.71073)	Unit cell dimensions [Å] and [°]	7.4978(3)	87.1053(15)
Crystal size / [mm^3]	0.21 × 0.088 × 0.061		10.7291(4)	75.2936(16)
Crystal habit	clear colourless block		12.8583(5)	85.7968(15)
Density (calculated) / [g/cm^3]	1.504	Absorption coefficient / [mm^{-1}]	0.496	
Abs. correction Tmin	0.617	Abs. correction Tmax	0.746	
Abs. correction type	multiscan	F(000) [e$^-$]	470	

Table S7. Data collection and structure refinement of **P-III**.

Index ranges	-10 ≤ h ≤ 10, -15 ≤ k ≤ 15, -18 ≤ l ≤ 18	Theta range for data collection [°]	4.942 to 60.17	
Reflections number	64560	Data / restraints / parameters	11621/3/507	
Refinement method	Least squares	Final R indices	all data	R1 = 0.0365, wR2 = 0.0853
Function minimized	Σ w(F_o^2 - F_c^2)^2		I>2σ(I)	R1 = 0.0324, wR2 = 0.0839
Goodness-of-fit on F^2	1.103	Weighting scheme	w=1/[σ^2(Fo^2)+(0.0571P)^2]	
Largest diff. peak and hole [e Å^{-3}]	0.38/-0.63		where P=(F_o^2+2F_c^2)/3	

Table S8. Sample and crystal data of (–)-monophyllidin polymorph **P-IV**.

Chemical formula	$C_{16}H_{26}NO_{8.5}$	Crystal system	triclinic	
Formula weight [g/mol]	368.38	Space group	*P1*	
Temperature [K]	100	Z	2	
Measurement method	\Φ and \ω scans	Volume [Å^3]	875.64(7)	
Radiation (Wavelength [Å])	MoKα (λ = 0.71073)	Unit cell dimensions [Å] and [°]	7.3954(3)	75.3504(15)
Crystal size / [mm^3]	0.172 × 0.167 × 0.091		10.7058(5)	72.7843(14)
Crystal habit	clear colourless needle		11.9675(5)	85.7711(14)
Density (calculated) / [g/cm^3]	1.397	Absorption coefficient / [mm^{-1}]	0.113	
Abs. correction Tmin	0.7024	Abs. correction Tmax	0.746	
Abs. correction type	multi-scan	F(000) [e$^-$]	394	

Table S9. Data collection and structure refinement of (–)-monophyllidin polymorph **P-IV**.

Index ranges	-10 ≤ h ≤ 10, -15 ≤ k ≤ 15, -16 ≤ l ≤ 16	Theta range for data collection [°]	5.768 to 60.15	
Reflections number	33632	Data / restraints / parameters	10051/4/483	
Refinement method	Least squares	Final R indices	all data	R1 = 0.0409, wR2 = 0.0943
Function minimized	Σ w(F_o^2 - F_c^2)^2		I>2σ(I)	R1 = 0.0357, wR2 = 0.0911
Goodness-of-fit on F^2	1.03	Weighting scheme	w=1/[σ^2(F_o^2)+(0.0595P)^2+0.0960P]	
Largest diff. peak and hole [e Å^{-3}]	0.37/-0.22		where P=(F_o^2+2F_c^2)/3	

Table S10. Sample and crystal data of (–)-monophyllidin polymorph **P-V**.

Chemical formula	$C_{16}H_{27}NO_9$	Crystal system	monoclinic	
Formula weight [g/mol]	377.38	Space group	*P21*	
Temperature [K]	100.0	Z	4	
Measurement method	\f and \w scans	Volume [Å3]	1793.5(2)	
Radiation (Wavelength [Å])	MoKα (λ = 0.71073)	Unit cell dimensions [Å] and [°]	7.0634(4)	90
Crystal size / [mm3]	0.1 × 0.09 × 0.03		30.387(2)	100.931(2)
Crystal habit	clear colourless block		8.5103(6)	90
Density (calculated) / [g/cm3]	1398	Absorption coefficient / [mm-1]	0.114	
Abs. correction Tmin	0,6238	Abs. correction Tmax	0,746	
Abs. correction type	multiscan	F(000) [e-]	808.0	

Table S11. Data collection and structure refinement of (–)-monophyllidin polymorph **P-V.**

Index ranges	0,6238	Theta range for data collection [°]	5.056 to 60.21	
Reflections number	79750	Data / restraints / parameters	10472/3/495	
Refinement method	Least squares	Final R indices	all data	R1 = 0.0508, wR2 = 0.0956
Function minimized	Σ w(F_o^2 - F_c^2)^2		I>2σ(I)	R1 = 0.0395, wR2 = 0.0875
Goodness-of-fit on F2	1040	Weighting scheme	w=1/[σ^2(Fo^2)+(0.0383P)^2 +0.4235P]	
Largest diff. peak and hole [e Å-3]	0.27/-0.24		where P=(Fo^2+2Fc^2)/3	

Table S12. Sample and crystal data of **P-VI**.

Chemical formula	$C_{16}H_{22}NO_{6.5}$	Crystal system	orthorhombic	
Formula weight [g/mol]	332.34	Space group	*P212121*	
Temperature [K]	100	Z	8	
Measurement method	\Φ and \ω scans	Volume [Å3]	3260.1(3)	
Radiation (Wavelength [Å])	MoKα (λ = 0.71073)	Unit cell dimensions [Å] and [°]	7.2055(4)	90
Crystal size / [mm3]	0.176 × 0.057 × 0.02		16.5857(7)	90
Crystal habit	clear colourless plate		27.2797(13)	90
Density (calculated) / [g/cm3]	1.354	Absorption coefficient/[mm-1]	0.105	
Abs. correction Tmin	0.6788	Abs. correction Tmax	0.746	
Abs. correction type	multi-scan	F(000) [e-]	1416	

Table S13. Data collection and structure refinement of **P-VI**.

Index ranges	-7 ≤ h ≤ 10, -17 ≤ k ≤ 23, -38 ≤ l ≤ 33	Theta range for data collection [°]	4.912 to 60.146	
Reflections number	30445	Data / restraints / parameters	9526/0/435	
Refinement method	Least squares	Final R indices	all data	R1 = 0.0716, wR2 = 0.1030
Function minimized	Σ w(Fo^2 - Fc^2)^2		I>2σ(I)	R1 = 0.0453, wR2 = 0.0951
Goodness-of-fit on F2	1.025	Weighting scheme	w=1/[σ^2(Fo^2)+(0.0553P)^2]	
Largest diff. peak and hole [e Å-3]	0.28/-0.36		where P=(Fo^2+2Fc^2)/3	

Table S14. Sample and crystal data of **5xHCl**.

Chemical formula	$C_{26}H_{40}ClNO_6$	Crystal system	orthorhombic	
Formula weight [g/mol]	498.04	Space group	*P212121*	
Temperature [K]	130	Z	4	
Measurement method	\Φ and \ω scans	Volume [Å3]	2798.4(11)	
Radiation (Wavelength [Å])	MoKα (λ = 0.71073)	Unit cell dimensions[Å] and [°]	11.151(3)	90
Crystal size / [mm3]	0.29 × 0.24 × 0.03		43.990(10)	90
Crystal habit	clear colourless plate		5.7048(13)	90
Density (calculated) / [g/cm3]	1.182	Absorption coefficient / [mm-1]	0.174	
Abs. correction Tmin	0.5784	Abs. correction Tmax	0.7460	
Abs. correction type	multi-scan	F(000) [e-]	1072	

Table S15. Data collection and structure refinement of **5xHCl**.

Index ranges	$-11 \le h \le 15$, $-61 \le k \le 61$, $-7 \le l \le 8$	Theta range for data collection [°]	4.59 to 60.028	
Reflections number	25481	Data / restraints / parameters	8075/0/314	
Refinement method	Least squares	Final R indices	all data	R1 = 0.1120, wR2 = 0.2049
Function minimized	$\Sigma\, w(F_o^2 - F_c^2)^2$		$I > 2\sigma(I)$	R1 = 0.0861, wR2 = 0.1935
Hydrogen Goodness-of-fit on F2	1.082	Weighting scheme	$w=1/[\sigma^2(F_o^2)+(0.0468P)^2+6.0740P]$	
Largest diff. peak and hole [e Å-3]	0.80/-0.34		where $P=(F_o^2+2F_c^2)/3$	

S5.2 Hydrogen bond geometries for (–)-monophyllidin P-I to P-V

Table S16. Bond length and angles of hydrogen bond networks revealed in (–)-monophyllidin polymorphs **P-II** to **P-VI**

	N1AH- O2A [Å,°]		N1AH-O3A [Å,°]		N1BH-O2B [Å,°]		N1HB-O6B [Å,°]	
P-II	2.067	111.259	2.371	118.005	1.937	118.050	2.235	123.555
P-III	1.918	118.089	2.794	112.547	1.877	119.308	2.237	124.259
P-IV	2.025	112.709	2.390	118.985	1.961	117.404	2.297	122.504
P-V	2.072	109.765	2.325	119.813	2.091	113.476	2.802	108.976
P-VI	1.940	116.883	2.376	119.373	1.987	115.181	2.576	114.322

Table S16. Hydrogen-bond geometry (Å, °) for (–)-monophyllidin polymorph **P-I**

D	H	A	d(D-H)/Å	d(H-A)/Å	d(D-A)/Å	D-H-A/°
O1	H1	N1	0.84	1.897	2.6561(11)	149.7
N2	H2	O1	0.88	2.015	2.7120(11)	135.3

Table S17. Hydrogen bonds geometry (Å, °) for (–)-monophyllidin polymorph **P-II**

D	H	A	d(D-H)/Å	d(H-A)/Å	d(D-A)/Å	D-H-A/°
O3A	H3A	O4A	0.84	1.71	2.458(2)	147.6
O3S	H3S	O4B[1]	0.87	1.96	2.820(2)	172.4
N1A	H1A	O2A	1.00	2.07	2.603(3)	111.3
N1A	H1A	O3A	1.00	2.37	2.975(2)	118.0
O3B	H3B	O4B	0.84	1.72	2.470(2)	148.2
N1B	H1B	O2B	1.00	1.94	2.564(3)	118.1

N1B	H1B	O6B	1.00	2.23	2.909(2)	123.6
O1S	H1SB	O2A	0.87	2.00	2.863(3)	172.3
O2S	H2S	O2B	0.87	2.01	2.859(3)	165.5

Symmetry code: [1]1-X,+Y,1-Z

Table S18. Hydrogen bonds geometry (Å, °) for (–)-monophyllidin polymorph **P-III**

D	H	A	d(D-H)/Å	d(H-A)/Å	d(D-A)/Å	D-H-A/°
O3B	H3B	O4B	0.84	1.73	2.484(2)	147.8
N1B	H1B	O2B	1.00	1.88	2.522(2)	119.3
N1B	H1B	O6B	1.00	2.24	2.919(2)	124.3
O3A	H3A	O4A	0.84	1.73	2.484(2)	147.8
N1A	H1A	O2A	1.00	1.92	2.546(2)	118.1
C8A	H8A	O6A	1.00	2.42	3.277(2)	143.1
O7	H7A	O2A[1]	0.87	1.93	2.794(3)	170.5
O7	H7B	O1B[2]	0.87	1.94	2.785(3)	162.6

Symmetry code: [1] 1+X, 1+Y, +Z; [2] +X, +Y, -1+Z

Table 19. Hydrogen-bond geometry (Å, °) for (–)-monophyllidin polymorph **P-IV**

D	H	A	d(D-H)/Å	d(H-A)/Å	d(D-A)/Å	D-H-A/°
O1	H1	N1	0.84	1.897	2.6561(11)	149.7
N2	H2	O1	0.88	2.015	2.7120(11)	135.3

Table 20. Hydrogen-bond geometry (Å, °) for (–)-monophyllidin polymorph **P-V**

D	H	A	d(D-H)/Å	d(H-A)/Å	d(D-A)/Å	D-H-A/°
O3A	H3A	O4A	0.84	1.69	2.446(2)	148.6
N1A	H1A	O3A	1.00	2.33	2.953(3)	119.8
O3B	H3B	O4B	0.84	1.70	2.459(2)	149.0
N1B	H1B	O2B	1.00	2.09	2.653(2)	113.5
N1B	H1B	O4S1	1.00	2.25	3.009(2)	131.7

Symmetry code: 1+X,+Y,-1+Z

Table 21. Hydrogen-bond geometry (Å, °) for (–)-monophyllidin polymorph **P-VI**

D	H	A	d(D-H)/Å	d(H-A)/Å	d(D-A)/Å	D-H-A/°
O3A	H3A	O4A	0,84	1,71	2,467(2)	148,2
N1A	H1A	O2A	1	1,94	2,553(3)	116,9
N1A	H1A	O3A	1	2,38	2,996(2)	119,4
O3B	H3B	O4B	0,84	1,7	2,457(2)	149,1

N1B	H1B	O2B	1	1,99	2,577(2)	115,2
N1B	H1B	O6B	1	2,58	3,123(2)	114,3
O7	H7A	O1A[1]	0,87	2,05	2,920(3)	173,3
O7	H7B	O2B[1]	0,87	1,96	2,824(3)	171,7

Symmetry code: [1]-1+X,+Y,+Z

Table 22. Hydrogen-bond geometry (Å, °) for (–)-monophyllidin-(+)-menthyl ester **5xHCl**

D	H	A	d(D-H)/Å	d(H-A)/Å	d(D-A)/Å	D-H-A/°
O1	H1	N1	0.84	1.897	2.6561(11)	149.7
N2	H2	O1	0.88	2.015	2.7120(11)	135.3
